# Asynchronous patterns in soil bacterial diversity and functional potentials along an alpine altitudinal gradient

**DOI:** 10.3389/fmicb.2024.1428815

**Published:** 2024-12-02

**Authors:** Xianping Li, Teng Li, Huixin Li

**Affiliations:** College of Resources and Environmental Sciences, Nanjing Agricultural University, Nanjing, China

**Keywords:** soil bacteria, altitudinal distribution, functional potential, co-occurrence network, phylogenetic diversity

## Abstract

**Introduction:**

Altitudinal changes in soil bacterial diversity, composition, biotic interactions, and function are prevalent. However, the overall patterns and associations among these dimensions remain unclear, particularly in vulnerable alpine mountain ecosystems.

**Methods:**

Here, we investigated soil bacterial communities along a high-altitude gradient to elucidate patterns and associations in taxonomic and phylogenetic diversity, co-occurrence networks, and functional potentials.

**Results:**

We observed increasing altitudinal trends in bacterial richness and phylogenetic diversity, along with significant differences in taxonomic and phylogenetic composition across altitudes. The connectivity component of the co-occurrence network properties showed a negative association with altitude. We also observed high redundancy in functional potentials, resulting in insignificant variation in functional diversity along the altitudinal gradient. However, the strength of functional diversity varied based on the interaction between network connectivity and phylogenetic diversity. Additionally, functional dissimilarity was more closely associated with phylogenetic rather than taxonomic dissimilarity or differences in network properties, highlighting the role of phylogenetic lineages in functional redundancy.

**Discussion:**

This study characterizes the altitudinal distribution of soil bacteria and explores their covariations, enhancing our understanding of soil bacterial diversity and functional potentials along altitudinal gradients and providing valuable insights for predicting community changes and improving alpine ecosystem conservation.

## 1 Introduction

Species responses to environmental gradients are multifaceted, encompassing composition, phylogeny, biotic interactions, and functioning. Understanding these ecological patterns and their interrelationships is crucial in both ecology and biogeography (Tilman et al., 2014; Oliver et al., 2015; Valiente-Banuet et al., 2015). Despite the growing body of knowledge on species’ spatial patterns, our understanding of covariation among these dimensions remains limited, particularly in less-explored environments such as high mountains and for functionally significant soil organisms like bacteria.

Soil bacteria are a critical component of soil biodiversity, characterized by their ubiquitous distribution, taxonomic diversity, and evolutionary antiquity. These microorganisms play pivotal roles in soil ecosystems by facilitating nutrient cycling, organic matter decomposition, plant growth promotion, and bioremediation, thus serving as a foundation for ecological research, biodiversity conservation, and ecosystem functioning maintenance (Bardgett and van der Putten, 2014; Banerjee and van der Heijden, 2022). Extensive research efforts have been dedicated to exploring the geographic distribution patterns of soil bacteria, with particular emphasis on their taxonomic and phylogenetic diversity and composition (Lozupone and Knight, 2007; Delgado-Baquerizo et al., 2018). Recent advancements in measurement, experimentation, sequencing, and analytical technologies have enabled precise elucidation and validation of bacterial community functions and interactions among community members (Barberan et al., 2012; Layeghifard et al., 2017; Geisen et al., 2019). However, the extent to which taxonomic and phylogenetic diversity and composition respond similarly to environmental changes, and how these differences influence bacterial biotic associations and functional potentials, remains inadequately understood.

Mountains, characterized by significant environmental variability over short distances, are recognized as terrestrial biodiversity hotspots and focal points of ecological research (Rahbek et al., 2019). They also represent frontiers in soil microbial research (Bryant et al., 2008; Fierer et al., 2011). It is hypothesized that taxonomic diversity decreases linearly or non-linearly with increasing altitude due to harsh environmental conditions (e.g., low temperatures) at higher altitudes (Rahbek, 1995; McCain and Grytnes, 2010). Conversely, phylogenetic diversity may parallel taxonomic diversity, reflecting similar ecological and evolutionary processes along altitudinal gradients (Dreiss et al., 2015; He et al., 2022). Alternatively, it may diverge from taxonomic diversity, resulting in scenarios such as low phylogenetic diversity despite high taxonomic diversity (e.g., taxa closely related within evolutionary lineages) or high phylogenetic diversity despite low taxonomic diversity (e.g., strong environmental filtering exerting selective pressures on taxa within lineages) (Zhang et al., 2020; Ding et al., 2021; Zhang et al., 2021). For soil bacteria, predominantly negative and non-significant correlations between taxonomic diversity and altitude have been observed (Bryant et al., 2008; Fierer et al., 2011; Shen et al., 2014; Wang et al., 2015; Ma et al., 2022). Similar patterns have been reported for bacterial phylogenetic diversity (Bryant et al., 2008; Wang et al., 2015; Zhang et al., 2018; Ma et al., 2022). However, the covariation between bacterial taxonomic and phylogenetic diversity and composition along altitudinal gradients has been scarcely addressed, leaving ecological and evolutionary processes concerning soil bacteria in mountainous regions undetermined.

The relationship between biodiversity and ecosystem functioning is a fundamental topic in ecology (Graham et al., 2016; Banerjee et al., 2018). High diversity, particularly high phylogenetic diversity, has been shown to promote and maintain ecosystem functioning through functional complementarity (Allison and Martiny, 2008; Srivastava et al., 2012). However, these positive correlations may vary depending on the context of soil bacterial communities (Nannipieri et al., 2003; Beier et al., 2017). For example, harsh environmental conditions, such as pollution, may lead to stronger correlations between community composition and functional potentials compared to benign conditions, as these conditions may select for specific taxa with particular functions, whereas benign conditions may support diverse taxa with redundant functions (Liu L. et al., 2021). Specifically, altitude-related factors, such as decreasing temperatures, may reduce soil bacterial metabolism and enzymatic activity (Xu et al., 2014; Feng et al., 2021; Ren et al., 2021). Additionally, cold-tolerant and poor-nutrient-tolerant taxa may be selected and enriched at higher altitudes (Feng et al., 2021). These changes may significantly alter the functional profiles of soil bacterial communities along altitudinal gradients, accompanied by notable variations in community composition. However, the relative effects of taxonomic and phylogenetic aspects on bacterial functional changes remain unclear.

Beyond changes in community composition, whether taxonomically or phylogenetically, biotic interaction patterns also transform along environmental gradients such as altitude. The commonly proposed biotic interaction hypothesis suggests a decrease in interaction strength with increasing altitude (or latitude) (Roslin et al., 2017; Zvereva and Kozlov, 2022), although considerable variability has been observed (Moles et al., 2011; Zvereva and Kozlov, 2022). However, this hypothesis may not fully apply to soil bacteria due to their potential for diverse interactions and high functional redundancy (Allison and Martiny, 2008; Faust and Raes, 2012; Louca et al., 2018). Directly investigating soil bacterial interactions poses challenges due to the extreme diversity, small size, and abundance of soil biota. Co-occurrence network analysis offers a feasible approach to unveil potential associations among soil bacterial taxa (Barberan et al., 2012; Deng et al., 2012). Studies along altitudinal gradients have revealed distinct bacterial co-occurrence patterns. For instance, Wu et al. (2024) observed an increase in network complexity and stability with altitude in stream bacterial communities, while Chen et al. (2022) reported a decline in the network complexity of soil bacterial communities with increasing altitude. General altitudinal patterns in bacterial co-occurrence require further exploration across different environments. Moreover, biotic interactions among bacterial community members have been identified as important drivers of soil functions, as evidenced by both laboratory and field studies (Wagg et al., 2019; Romdhane et al., 2022). Some studies suggested that interaction effects may have a more direct and stronger impact on ecosystem function than diversity or composition (Chen et al., 2022). Further assessment of the relative significance of co-occurrence patterns on soil bacterial functional potential along altitudinal gradients is still needed.

The Tibetan Plateau stands out for its unique biota, environmental conditions, and sensitivity to climate change, making it a focal point for biodiversity research and conservation efforts (Xu et al., 2009; Deng et al., 2020). Several studies have focused on soil bacteria in this region, revealing diverse patterns in diversity and composition along altitudinal gradients across different mountain ranges (Xu et al., 2014; Wang et al., 2015; Shen et al., 2019; Feng et al., 2021). However, the relationships between bacterial diversity, community structure, functional potentials, and biotic associations remain largely unexplored, hindering a comprehensive understanding of soil bacterial processes in the Tibetan Plateau.

In this study, we investigated the distribution of soil bacterial communities using high-throughput sequencing along an altitudinal gradient in Balang Mountain on the eastern edge of the Tibetan Plateau. Our objectives were to (1) characterize the taxonomic and phylogenetic diversity and composition patterns of soil bacteria and their consistency along altitudes, (2) quantify the structure of biotic interactions within bacterial communities using co-occurrence network analysis, and (3) determine soil bacterial functional potentials and explore their associations with the taxonomic, phylogenetic, and co-occurrence structures of bacterial communities.

## 2 Materials and methods

### 2.1 Study site and soil sampling

Our investigation took place in Balang Mountain (102.86°E, 30.96°N), located within the Wolong Nature Reserve, Sichuan Province, China, spanning an altitude range of 3,136 to 4,128 m. This region features a subtropical monsoon moist climate, with a mean annual temperature ranging from −0.57 to 4.62°C and annual precipitation varying from 709 to 790 mm along the sampling elevation gradient, according to WorldClim Version 2^1^.

Soil sampling was conducted in August 2022. Six sampling sites were strategically selected along the altitudinal gradient. These sites were located on gentle slopes, each comprising six plots spaced approximately 10 m apart. The sampling procedure involved the initial removal of litter, roots, and stones. Subsequently, we collected five soil cores from each sampling plot, positioned at the four corners and the center within a 1 m × 1 m square. Each core measured 5 cm in diameter and extended to a depth of 10 cm. To ensure homogeneity, soil cores from each plot were thoroughly mixed. The soil was then sieved through 2 mm meshes and stored in a portable dry ice container for molecular analyses. In total, 36 soil samples were gathered across the altitudinal gradient.

### 2.2 Amplicon sequencing and bioinformatic analyses

Soil bacterial DNA extraction was performed using HiPure Soil DNA Kits (Magen, Guangzhou, China) following the manufacturer’s protocols. PCR amplification of the 16S rDNA V3-V4 region of the ribosomal RNA gene was performed under the following conditions: 95°C for 5 min, followed by 30 cycles at 95°C for 1 min, 60°C for 1 min, and 72°C for 1 min, with a final extension at 72°C for 7 min. The primers used were 341F (5’-CCTACGGGNGGCWGCAG-3′) and 806R (5’-GGACTACHVGGGTATCTAAT-3′). Amplicons were extracted from 2% agarose gels and purified using the AxyPrep DNA Gel Extraction Kit (Axygen Biosciences, Union City, CA, United States) following the manufacturer’s instructions. The purified amplicons were pooled equimolarly and subjected to paired-end sequencing (PE250) on an Illumina platform according to standard protocols.

The DADA2 pipeline was employed to process paired-end fastq files, yielding merged, denoised, chimera-free, and inferred sample sequences (Callahan et al., 2016). Briefly, raw reads were filtered and truncated by removing reads containing unknown nucleotides and primer sequences. A dereplicated list of unique sequences and their abundances, along with consensus positional quality scores, was generated. Paired-end denoised reads were merged into raw ASVs (amplicon sequence variants) with a minimum overlap of 12 bp. After chimera removal, denoised, chimera-free ASV sequences and their abundances were obtained.

The representative ASV sequences were classified using a naive Bayesian model with the RDP classifier (Wang et al., 2007) based on the SILVA database (Pruesse et al., 2007). A phylogenetic tree was constructed using FastTree (Price et al., 2010). Only ASVs present in more than two samples were included in subsequent analyses to minimize noise and reduce false-positive predictions. ASVs were normalized by scaling with ranked subsampling to preserve the original community structure by minimizing subsampling errors (Beule and Karlovsky, 2020).

### 2.3 Bacterial diversity, co-occurrence network, and functional potential estimation

The taxonomic diversity of the bacterial communities was characterized using richness and Shannon diversity metrics with the R package vegan (Oksanen et al., 2024). Phylogenetic diversity (PD) was assessed using Faith’s index (an unweighted index) and its abundance-weighted version (Faith, 1992; Barker, 2002). Phylogenetic diversity typically correlates positively with richness (Swenson, 2014). To account for the effect of richness on PD, standardized effect sizes (SES) of PD were calculated by shuffling taxa labels across tips of the phylogeny with 999 randomizations. The unweighted Faith’s index was calculated using the R package picante (Kembel et al., 2010), while the weighted version was calculated following the code provided by Swenson (2014). Differences in bacterial community composition among altitudes were evaluated using the Bray–Curtis index on Hellinger-transformed data for taxonomic data with the R package vegan (Legendre and Gallagher, 2001) and the weighted UniFrac distance for phylogenetic data with the R package GUniFrac (Chen et al., 2012).

The meta-community co-occurrence network was inferred using the statistical method SpiecEasi, which has been shown to outperform other methods (e.g., SparCC, CCREPE, and Pearson correlation) for microbial ecological network inference (Kurtz et al., 2015). ASVs present in less than half of the samples were excluded to ensure reliable association inference and to better characterize the core bacterial community members (Yuan et al., 2021). The bacterial composition data were first centered log-ratio transformed and then subjected to a graphical model inference procedure of neighborhood selection, followed by stability-based model selection, to infer microbial associations (Kurtz et al., 2015). Sub-networks of each bacterial community were generated from the meta-community network by preserving ASVs present in each community, as described by Ma et al. (2016). Twenty widely used network-level topological properties were calculated for each sub-network to illustrate the structure of microbial networks (Supplementary Table S1).

The functional potentials of each bacterial community were predicted using PICRUSt2 based on marker gene sequencing profiles (Douglas et al., 2020). PICRUSt2 is known for its accuracy and flexibility in bacterial functional predictions compared to its previous version and other competing methods (Douglas et al., 2020). It utilizes ASV sequences and abundances as input and outputs pathway abundances based on the Kyoto Encyclopedia of Genes and Genomes (KEGG) database (Douglas et al., 2020). The diversity of functional potentials was estimated using the Shannon index with the R package vegan, considering both the richness and evenness of the pathways. Functional differences among communities were quantified using the Hellinger-transformed Bray–Curtis index based on KEGG pathway abundance with the R package vegan. We focused on predicted functional potentials rather than measured soil functions, as the former is more directly connected to soil bacteria, while the latter can be influenced by various soil biotas simultaneously. However, it is important to note that predictions can be biased due to the biased existence of reference genomes, and their accuracy may vary across sample types and functional categories (Douglas et al., 2020; Sun et al., 2020; Djemiel et al., 2022). Additionally, linear discriminant analysis (LDA) effect size (LEfSe) was used to identify differentially abundant functional and taxonomic features (false discovery rate-adjusted *p* < 0.05) across altitudes using the R package microeco (Segata et al., 2011; Liu C. et al., 2021).

### 2.4 Statistical analyses

Altitudinal trends in taxonomic, phylogenetic, and functional diversities were assessed using the linear regression models. Principal component analysis (PCA) was performed to reduce redundancy among the 20 topological properties obtained from network analysis. Network structure was characterized by the first two principal components, which explained the most variation in network properties. These components were then regressed against altitude to examine variations in network structure along the altitudinal gradient. The relationships between taxonomic diversity, phylogenetic diversity, network structure, and functional diversity were assessed using Pearson correlation coefficients. A multiple linear model was constructed with taxonomic diversity (Shannon diversity), phylogenetic diversity (abundance-weighted Faith’s index), and network structure (the first two independent principal components) as independent variables, and soil bacterial functional diversity as the dependent variable. These variables were chosen to maintain model simplicity while incorporating comprehensive variables with low pairwise correlations (Pearson’s correlation *r* < 0.7; variance inflation factors <3; Supplementary Tables S2, S3). All variables were standardized before modeling. The relative importance of each predictor was evaluated using dominance analysis, which decomposes the total explained variation (*R*^2^) into different parts explained by each variable in the model. This approach quantifies the changes in *R*^2^ of the regression model from adding one predictor to all possible combinations of the other predictors (Budescu, 1993). The relative contribution of each predictor was determined based on the average increase in *R*^2^ across all possible orderings using the R package relaimpo (Groemping, 2006).

Differences in bacterial taxonomic, phylogenetic, and functional composition, as well as network properties among altitudes, were tested using PERMANOVA with 999 permutations in the R package vegan (Anderson, 2001). Spearman correlations among distances (or dissimilarities) of taxonomic (Bray–Curtis index), phylogenetic (weighted UniFrac index), functional (Bray–Curtis index), and network (Euclidean index) dimensions were assessed using the Mantel test with 999 permutations in the R package vegan. Additionally, the composition differences in the ordination spaces (PCoA for taxonomic, phylogenetic, and functional dissimilarities, and PCA for network property distances) were tested and visualized using symmetric Procrustes analysis with the R package vegan (Peres-Neto and Jackson, 2001). Multiple regression on distance matrices (MRM), an extension of partial Mantel analysis, was used to estimate the effects of taxonomic dissimilarity, phylogenetic dissimilarity, and distance in network topological properties on the dissimilarity of functional potentials using the R package ecodist (Goslee and Urban, 2007; Lichstein, 2007). The total variation in the dissimilarity of functional potentials was decomposed into the unique contributions of the three distances/dissimilarities, their shared effects, and undetermined components to demonstrate the relative contributions of different factors (Borcard et al., 1992; Tuomisto and Ruokolainen, 2006). Briefly, MRM models were constructed with different combinations of predictors to assess the individual and shared effects of taxonomic dissimilarity, phylogenetic dissimilarity, and network topological distance.

All analyses were conducted in R version 4.3.1 (R Core Team, 2023).

## 3 Results

### 3.1 Bacterial diversity and composition

**Figure 1:**
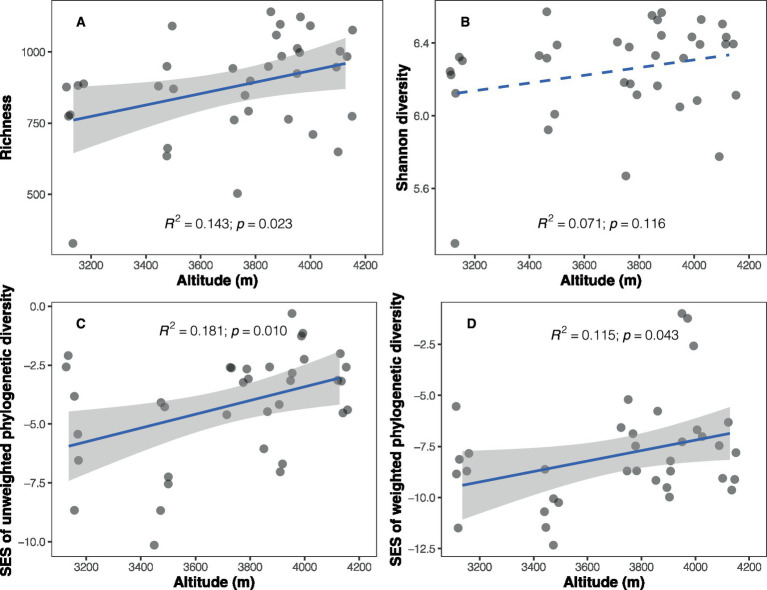
Altitudinal trends in soil bacterial taxonomic and phylogenetic diversity. **(A)** Richness; **(B)** Shannon diversity; **(C)** standardized effect size of unweighted phylogenetic diversity (Faith’s index); **(D)** standardized effect size of weighted phylogenetic diversity (weighted Faith’s index). Solid blue lines represent significant relationships between diversity and altitude based on a simple linear regression model, while dashed line indicates a non-significant relationship.

A total of 237,888 high-quality sequences representing soil bacteria were obtained and clustered into 8,279 bacterial ASVs. Bacterial richness (number of ASVs) ranged from 328 to 1,141 among the samples, exhibiting a positive trend with increasing altitude (Pearson’s *r* = 0.378, *p* = 0.023; Figure 1A and Supplementary Table S2). However, no significant altitudinal trend was observed for bacterial Shannon diversity (*r* = 0.267, *p* = 0.116; Figure 1B). The observed soil bacterial phylogenetic diversity, whether abundance-weighted (*r* = 0.501, *p* = 0.002) or unweighted (*r* = 0.498, *p* = 0.002), showed a positive correlation with altitude (Supplementary Table S3). Given the significant correlation between phylogenetic diversity and richness (unweighted PD: *r* = 0.948, *p* < 0.001; weighted PD: *r* = 0.800, *p* < 0.001; Supplementary Figure S1), SES values of phylogenetic diversity were calculated to disentangle the impact of richness. The results revealed positive relationships between SES values of PD and altitude (unweighted PD: *r* = 0.425, *p* = 0.010; weighted PD: *r* = 0.340, *p* = 0.043; Figures 1C,D).

**Figure 2:**
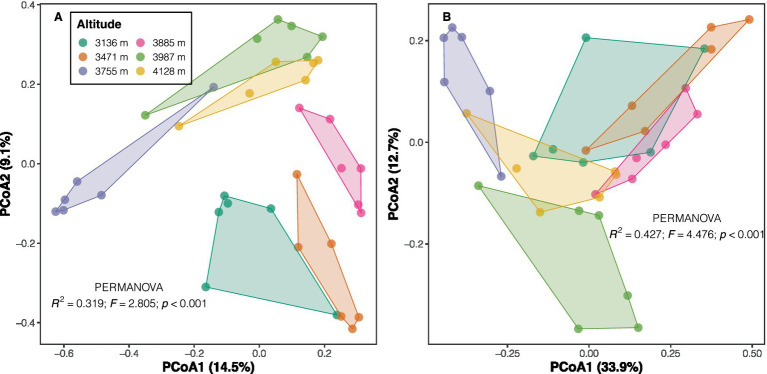
PCoA plots of taxonomic **(A)** and phylogenetic **(B)** community composition across altitudes. Taxonomic composition was characterized using the Bray–Curtis index on Hellinger-transformed data, while phylogenetic composition was based on weighted UniFrac distance. Inset text shows the results of the PERMANOVA test.

*Proteobacteria* emerged as the dominant phylum across altitudes (with a mean relative abundance of 31.3%), followed by *Acidobacteria* (22.8%), *Verrucomicrobia* (11.4%), and *Bacteroidetes* (10.2%). Other phyla exhibited relative abundances below 10% (Supplementary Figure S2A). Overall, high altitudes showed greater abundances of phyla such as *Patescibacteria* and *Actinobacteria*, as well as classes like *Parcubacteria* and *Ignavibacteria*, compared to low altitudes, according to LEfSe results (LDA scores >2, *p* < 0.05; Supplementary Table S4 and Supplementary Figure S2B). In contrast, communities at low altitudes displayed significantly higher abundances of the phyla *Planctomycetes* and *Fusobacteria*, and classes like *Phycisphaerae*, *Bacilli*, and *Actinobacteria* (Supplementary Table S4 and Supplementary Figure S2B). PERMANOVA results indicated significant differences in both taxonomic (*F* = 2.805, *p* < 0.001) and phylogenetic compositions (*F* = 4.476, *p* < 0.001) among altitudes for soil bacterial communities (Figure 2). The Mantel test revealed a strong positive correlation between soil bacterial taxonomic and phylogenetic dissimilarities (Spearman’s *ρ* = 0.835, *p* < 0.001; Supplementary Figure S3A), a finding further confirmed by Procrustes analysis (e.g., smaller sum of squares, *m*^2^ = 0.151, *p* < 0.001; Supplementary Figure S3B).

### 3.2 Bacterial co-occurrence patterns

**Figure 3:**
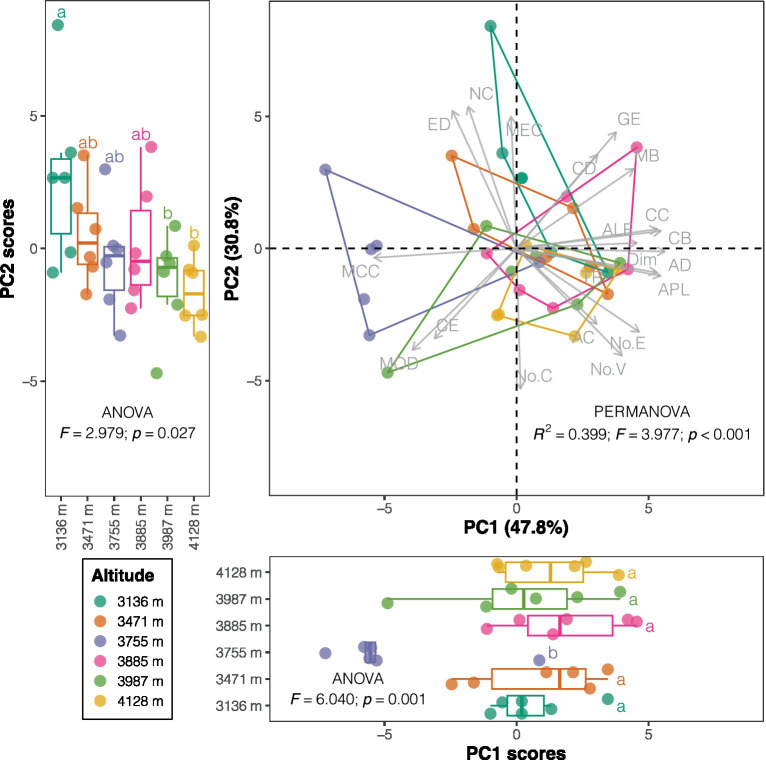
Principal component analysis (PCA) plot of co-occurrence network properties. The boxplots at the bottom and left represent the first and second principal component scores across altitudes, respectively. Inset text displays the results of the ANOVA test, and lowercase letters in the boxplots represent Tukey *post hoc* test results. Please refer to Supplementary Table S1 for the full names of each property.

A significant difference in the co-occurrence network structures of bacterial communities was observed across altitudes (PERMANOVA: *F* = 3.977, *p* < 0.001; Figure 3 and Supplementary Figure S4). Most of the measured co-occurrence network properties did not show significant trends along the altitudinal gradient (Supplementary Figure S5). However, the number of vertices, edges, and clusters positively correlated with altitude, while edge density, global efficiency, natural connectivity, and mean eigenvector centrality negatively correlated with altitude (all *p* < 0.05; Supplementary Figure S5). Given the high correlations among these network properties (Supplementary Figure S6), PCA was performed to reduce redundancy. The first and second principal components explained 47.8 and 30.8% of the total variation in network properties, respectively (Figure 3 and Supplementary Figure S7). Network properties with high loadings on the first principal component, such as average degree, centralization (betweenness), average path length, centralization (closeness), and mean closeness centrality, were generally not related to altitude (Supplementary Figures S5, S7). In contrast, altitude-related network properties, including natural connectivity, edge density, mean eigenvector centrality, and the number of clusters, exhibited high loadings on the second principal component (Supplementary Figures S5, S7). The different contributions of these network properties to the principal components and their varied responses to altitude resulted in distinct altitudinal trends for the first two principal component scores: the scores of the first principal component (*r* = 0.018, *p* = 0.917) showed no relationship with altitude, while those of the second principal component (*r* = −0.547, *p* < 0.001) were negatively correlated with altitude (Figure 3 and Supplementary Figure S8). Therefore, the structure of soil bacterial co-occurrence networks along the altitudinal gradient could be effectively represented by two orthogonal components (i.e., PC1 and PC2).

### 3.3 Bacterial functional potentials

**Figure 4:**
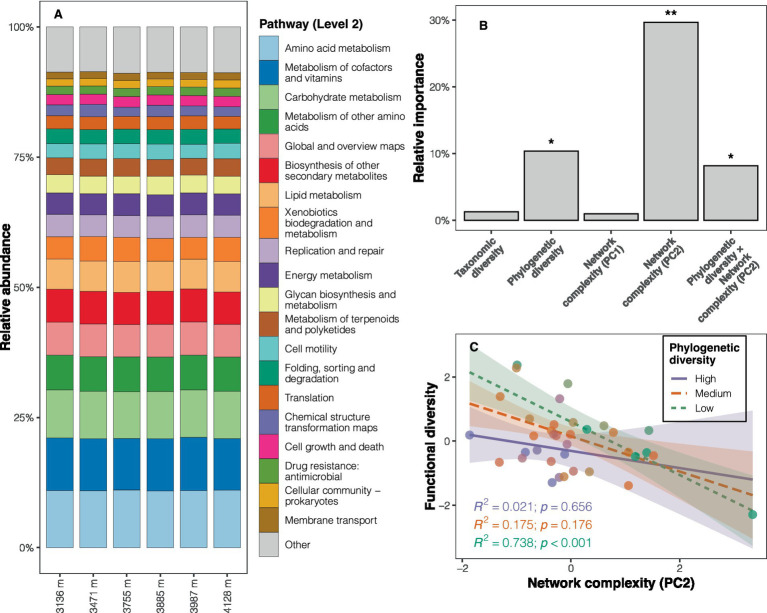
Composition of soil bacterial functional potentials and determinants of the diversity of functional potentials. **(A)** Relative abundance of KEGG pathways at Level 2 across altitudes. **(B)** Results of the dominance analysis, with the significance of each predictor determined by the multiple regression model (*p < 0.05, **p < 0.01). **(C)** Interaction plot depicting the relationship between network complexity (PC2) and functional diversity at different levels of phylogenetic diversity, controlling for other predictors, to illustrate the interaction effect in the regression model. The three levels (high, medium, and low) correspond to phylogenetic diversity values greater than 1 SD (standard deviation), between −1 SD and 1 SD, and less than −1 SD, respectively.

The functional predictions revealed that metabolism was the dominant pathway at Level 1 (relative abundance: 73.7%; Supplementary Figure S9A), with amino acid metabolism (11.0%), metabolism of cofactors and vitamins (10.0%), and carbohydrate metabolism (9.1%) being the predominant pathways at Level 2 (Figure 4A). KEGG pathways such as energy metabolism, the endocrine system, and replication and repair (e.g., mismatch repair, homologous recombination) were more abundant at high altitudes (LDA scores >2, *p* < 0.05) compared to low altitudes (Supplementary Table S5 and Supplementary Figure S9B). Pathways with significantly higher abundance at low altitudes included carbohydrate metabolism, glycan biosynthesis and metabolism, metabolism of other amino acids, and nucleotide metabolism (Supplementary Table S5 and Supplementary Figure S9B). Although functional diversity (based on Level 3) showed no significant altitudinal trend (Supplementary Figure S10A), the composition of soil bacterial functional potentials differed among altitudes (PERMANOVA: *F* = 3.220, *p* < 0.001; Supplementary Figure S10B).

The correlation between phylogenetic diversity and functional diversity changed when including the variable representing the structure of co-occurrence networks (PC2) in the multiple regression models (Supplementary Table S6). Consequently, an interaction term (phylogenetic diversity × PC2) was added to the model to account for the moderation effect of PC2 on the relationship between phylogenetic diversity and functional diversity. The updated regression model exhibited superior performance (*R*^2^_adj_ = 0.422, AICc = 93.8, *F* = 6.119, *p* < 0.001) compared to other candidate models (Supplementary Table S6). This model highlighted the significance of phylogenetic diversity, PC2, and their interaction term as predictors of functional diversity (all *p* < 0.05; Supplementary Table S6 and Figure 4B). The dominance analysis further revealed that PC2 accounted for a substantial portion of the variation in functional diversity (29.7%), followed by phylogenetic diversity (10.4%), and the interaction term (8.2%) (Figure 4B). Given the significant interaction effect between PC2 and phylogenetic diversity, an interaction plot was drawn for visualization. The interaction plot indicated that the negative correlation between network structure (PC2) and functional diversity diminished with increasing phylogenetic diversity (Figure 4C).

**Figure 5:**
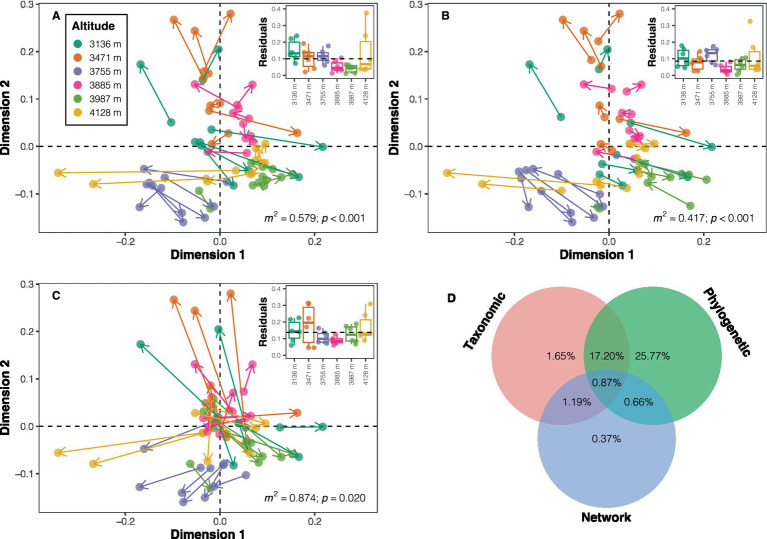
Visualization of similarity between ordinations using symmetric Procrustes analysis. Panels **(A–C)** depict Procrustes plots between functional potential ordination and ordinations of taxonomic composition, phylogenetic composition, and co-occurrence network properties, respectively. The beginnings of the segments indicate the position of the samples in the functional ordination, while the ends (arrows) of the segments point to the target ordinations. The inset plots display the residuals for each sample in each ordination comparison across altitudes. *m*^2^ denotes the sum of squares, with significance determined using permutation tests. **(D)** Contributions of different distances/dissimilarities to the dissimilarity in functional potentials.

Three approaches were employed to explore the influence of taxonomic dissimilarity, phylogenetic dissimilarity, and distance in network properties on variations in the dissimilarity of soil bacterial functional potentials. Firstly, the functional dissimilarity (mean value ± standard deviation: 0.020 ± 0.008) based on functional potentials was notably smaller than taxonomic dissimilarity (0.795 ± 0.073) and phylogenetic dissimilarity (0.271 ± 0.062). The Mantel test results suggested positive correlations between all three distances (or dissimilarities) and functional dissimilarity (taxonomic dissimilarity: *ρ =* 0.533; phylogenetic dissimilarity: *ρ =* 0.732; distance in network properties: *ρ =* 0.228; all *p* < 0.05; Supplementary Figure S11). Procrustes analyses demonstrated a higher similarity between phylogenetic dissimilarity and functional dissimilarity (*m*^2^ = 0.417, *p* < 0.001) compared to taxonomic dissimilarity (*m*^2^ = 0.579, *p* < 0.001) or distance in network properties (*m*^2^ = 0.874, *p* = 0.020) with functional dissimilarity in the ordination space (Figures 5A–C). A heuristic variation partitioning based on MRM further revealed that phylogenetic dissimilarity contributed the most to the variation in functional dissimilarity (unique effect: 25.77%; total effect: 44.50%), followed by taxonomic dissimilarity (unique effect: 1.65%; total effect: 20.91%), and distance in network properties (unique effect: 0.37%; total effect: 3.09%) (Figure 5D).

## 4 Discussion

Understanding how soil communities respond to environmental gradients across various ecological dimensions is complex yet insightful. In this study, we examined the altitudinal patterns of soil bacteria, a key component of soil biota, considering taxonomic, phylogenetic, co-occurrence, and functional aspects in an alpine mountain. Our findings reveal an increasing trend in both bacterial taxonomic and phylogenetic diversity with altitude, accompanied by a general decreasing trend in co-occurrence network connectivity. Additionally, although soil bacterial communities showed high redundancy in their functional potentials, we identified network connectivity as more closely related to functional diversity, a relationship influenced by phylogenetic diversity. Furthermore, our results indicated that the dissimilarity in bacterial functional potentials was more closely associated with phylogenetic structure than with taxonomic dissimilarity or variations in co-occurrence network properties.

Our case study demonstrated an increasing trend in soil bacterial diversity along altitude, both in terms of taxonomic and phylogenetic dimensions. This finding contrasts with the general hypothesis derived mainly from aboveground taxa (Rahbek, 1995; McCain and Grytnes, 2010) and some microbial studies (Bryant et al., 2008; Wang et al., 2015; Nottingham et al., 2018), which suggests a decrease in diversity with increasing altitude. However, similar patterns have been detected in other empirical microbial studies. For instance, microbial richness was found to be lower in subalpine soils (around 2,200 m) compared to alpine soils (around 2,600 m) in the central Alps (D'Alò et al., 2022); Wang et al. (2011) found that bacterial richness monotonously increased along altitude in a stony stream in Laojun Mountain (Wang et al., 2011); Shen et al. (2020) observed that bacterial diversity could increase at high altitudes (above 2,850 m) on Mt. Kilimanjaro (although the overall pattern was U-shaped) (Shen et al., 2020). This phenomenon may be attributed to the adaptability of soil bacteria to different environmental conditions compared to macro-organisms like plants and animals, which may promote taxa differentiation and coexistence in extreme conditions, thus contributing to higher diversity (Jansson and Taş, 2014; Donhauser and Frey, 2018). Nonetheless, the accumulating evidence of distinct altitudinal patterns in soil bacterial communities underscores the importance of further mechanistic exploration of microbial distribution.

Our results suggested that soil bacterial taxonomic and phylogenetic diversity generally covaried along altitude, as evidenced by the positive pairwise correlations and similar altitudinal trends. This finding aligns with previous studies across various taxonomic groups (Devictor et al., 2010; Safi et al., 2011; Arnan et al., 2017), suggesting that a greater number of species corresponds to a greater number of phylogenetic lineages. This phenomenon may be attributed to random sampling alone, wherein increased taxonomic richness is associated with increased phylogenetic diversity (Swenson, 2014). However, even after accounting for the effects of richness on phylogenetic diversity using random simulations, significant altitudinal trends in phylogenetic diversity persisted, underscoring the importance of evolutionary processes in shaping soil bacterial communities along altitudinal gradients. It is also important to note that some studies focused on soil bacteria along altitudinal gradients have found a divergence between taxonomic and phylogenetic diversity (Shen et al., 2015), indicating that environmental heterogeneity and the range of the altitudinal gradient may affect different diversity dimensions in varying ways.

The results of the co-occurrence network analysis revealed that the variation in network topological properties along the altitudinal gradient depended on the specific property examined. While the number of nodes and edges was positively correlated with altitude, largely due to increased richness, edge density, global efficiency, natural connectivity, and mean eigenvector centrality were significantly negatively correlated with altitude. These findings suggest a general reduction in the connectivity and complexity of soil bacterial co-occurrence networks with increasing altitude, a conclusion consistent with previous studies (Chen et al., 2022), implying a potential increase in the vulnerability of bacterial communities at high altitudes. These findings align with predictions that factors such as cold temperatures and shifts in pH at high altitudes can slow down metabolic processes or alter community composition, potentially weakening interactions within microbial networks (Li et al., 2020; Chen et al., 2022). However, direct and detailed assessments of soil bacterial interactions and their drivers along environmental gradients, both in field studies and controlled experiments, are crucial for understanding how soil diversity is organized and how it may respond to environmental change.

Although the diversity of soil bacterial functional potentials did not covary with altitude, its variation could be mediated by the interaction between network structure and phylogenetic diversity. Specifically, our study found a positive interaction effect between network connectivity (i.e., the second component of PCA on co-occurrence network properties) and phylogenetic diversity, alongside a negative effect of network connectivity on functional diversity. These findings generally contrast with previous findings and hypotheses suggesting that soil network complexity promotes ecosystem functions and/or enhances the positive effects of soil biodiversity on ecosystem functions (Wagg et al., 2019; Chen et al., 2022; Jiao et al., 2022). Several factors could explain these differences. Firstly, we focused on the functional potentials of soil bacteria rather than direct soil measurements related to nutrient provisioning, element cycling, or enzyme activities, which are influenced by interactions among multiple taxonomic groups under specific abiotic and biotic conditions over a relatively long time. Additionally, the positive relationship between network complexity and ecosystem functions remains uncertain, as its reliability varies depending on the taxon, biome, and conditions studied. For instance, soil fungal network complexity was negatively correlated with soil functions in tropical rainforest soils, whereas soil bacterial network complexity had a positive effect (Chen et al., 2023). A pot experiment also demonstrated that both bacterial and fungal network complexity negatively correlated with ecosystem functions under increasing ozone levels (Li et al., 2022). In our study, the negative effect of network structure (connectivity) on the diversity of functional potentials may be attributed to redundancy in soil microbial communities (Louca et al., 2018), as multiple interactions can fulfill similar functional potentials. Although phylogenetic diversity showed a weak correlation with functional diversity, its inclusion in the multiple regression model improved the model’s performance because it was correlated with network structure (connectivity). Therefore, phylogenetic diversity may act as a suppressor variable here (MacKinnon et al., 2000; Pandey and Elliott, 2010), suppressing the relationship between network connectivity and functional diversity. The redundancy or functional overlap among distant lineages may contribute to the low contribution of phylogenetic diversity to functionality, as the overall impact of individual taxa on functional potentials may be reduced, and the complementarity in functional roles among taxa is limited, particularly in communities with low network connectivity.

From a compositional perspective, differences in taxonomic and phylogenetic composition, as well as network properties, were all correlated with the composition of functional potentials. However, compared to taxonomic composition, phylogenetic and functional compositions exhibited less variation, particularly the functional composition, suggesting low functional complementarity or high functional redundancy between sites (Villéger et al., 2013; Heino and Tolonen, 2017). Nevertheless, the variation in the composition of functional potentials was better explained by phylogenetic dissimilarity than taxonomic dissimilarity, indicating that the functional roles of bacterial taxa may involve phylogenetically related taxa without necessarily being taxonomically specific (Jia and Whalen, 2020).

Several limitations may influence the robustness of the findings obtained in this study. Unlike the macro-taxa, where biotic interactions can be directly observed, interactions among microbial taxa are challenging to determine and are largely inferred based on static community composition using tools like network analysis. Consequently, the detected associations may not fully capture the true interactions among microbial taxa, particularly as interactions across multiple trophic groups that can affect soil diversity and functioning are often overlooked in such analyses. Nonetheless, co-occurrence network analysis remains a promising approach for assessing potential associations among microbial members at this stage. Additionally, accurately assessing soil functions and matching them to specific microbial groups remains challenging in microbial research. As a compromise, we focused on predicted functional potentials. While this approach may be conservative, as it cannot detect obvious changes in functions due to high functional redundancy, it offers broad coverage of potential functions that may emerge under changing environmental conditions. Furthermore, in this study, we focused on the patterns and covariations of different dimensions of soil bacterial communities across altitudes, leaving the underlying mechanisms and determinants unexplored. We anticipate that future studies will address this gap.

In conclusion, our study suggests that high altitudes may not necessarily constrain the diversity of soil bacteria taxonomically or phylogenetically. In contrast, the diversity of functional potentials appeared to be comparable across altitudes, likely due to strong functional redundancy among bacterial members. This finding implies generally robust resilience, resistance, and stability of bacterial functional potentials in high mountains and under future climate change scenarios. However, it is important to note that the diversity and composition of functional potentials may still be influenced by both the connectivity of the co-occurrence network and the phylogenetic structure of the bacterial community. This indicates that significant or accumulated changes in bacterial co-occurrence patterns and phylogenetic composition could still have considerable impacts on soil functions.

## Data Availability

The datasets presented in this study can be found in online repositories. The names of the repository/repositories and accession number(s) can be found: https://www.ncbi.nlm.nih.gov/, PRJNA1105430.

## Footnotes

^1^ https://www.worldclim.com
