# Supplementary material for: Asynchronous patterns in soil bacterial diversity and functional potentials along an alpine altitudinal gradient
Source: Front Microbiol. 2024 Dec 2;15:1428815. doi: 10.3389/fmicb.2024.1428815 (PMC11647016; doi:10.3389/fmicb.2024.1428815)
Supplement: Supplementary file 1 [file Data_Sheet_1.PDF]

Supplementary Material

Supplementary Table 1. Twenty co-occurrence network properties with abbreviation and short description.

**Name**	**Abbreviation**	**Description**
Number of vertices	No.V	Number of vertices of a graph
Number of edges	No.E	Number of edges of a graph
Edge density	ED	The ratio of the actual number of edges and the largest possible number of edges in the graph
Average degree	AD	Mean number of neighbors in the graph
Transitivity	TR	A measure of the probability that the adjacent vertices of a vertex are connected
Average path length	APL	Average path length in a graph, by calculating the shortest paths between all pairs of vertices
Global efficiency	GE	It is defined as the average of inverse distances between all pairs of vertices
Average local efficiency	ALE	Arithmetic mean of the local efficiencies across all the vertices
Assortativity coefficient	AC	It measures the level of homophyly of the graph, based on some vertex labeling or values assigned to vertices
Natural connectivity	NC	It is a useful robustness measure of complex networks, corresponding to the average eigenvalue of the adjacency matrix
Diameter	Dim	The length of the longest path of a graph
Number of clusters	No.C	Number of the maximal connected components of a graph
Centralization (degrees)	CD	A graph level centralization measure from the degree centrality scores of the vertices
Centralization (betweenness)	CB	A graph level centralization measure from the betweenness centrality scores of the vertices
Centralization (closeness)	CC	A graph level centralization measure from the closeness centrality scores of the vertices
Centralization (eigenvector)	CE	A graph level centralization measure from the eigenvector centrality scores of the vertices

Mean betweenness centrality	MB	Mean value of betweenness centrality
Mean closeness centrality	MCC	Mean value of closeness centrality
Mean eigenvector centrality	MEC	Mean value of eigenvector centrality
Modularity	MOD	Modularity score of a graph with respect to the community structure via greedy optimization

Supplementary Table 2. Taxonomic, phylogenetic, and functional diversity, as well as co-occurrence network properties, of soil bacterial communities across altitudes. Please refer to Supplementary Table 1 for the abbreviation of each network property.

Altitude	Plot	Taxonomic richness	Taxonomic Shannon diversity	Faith's index	SES of Faith's index	Weighted Faith's index	SES of weighted Faith's index	Functional Shannon diversity	No.V	No.E	ED	AD	TR	APL	GE	ALE	AC	NC	MB	Dim	No.C	CD	CB	CC	CE	MCC	MEC	MOD	PC1 of network properties	PC2 of network properties
3136 m	1	877	6.323	187.899	-2.575	137.207	-8.129	4.983	23	19	0.075	1.652	0.130	2.671	0.159	0.062	0.101	0.039	0.025	6	5	0.152	0.164	0.901	0.872	0.579	0.204	0.643	0.200	2.664
3136 m	2	328	5.298	93.960	-2.093	70.964	-5.538	4.967	14	11	0.121	1.571	0.000	2.194	0.207	0.089	-0.078	0.064	0.034	5	4	0.187	0.122	0.704	0.767	0.686	0.343	0.517	-0.987	8.420
3136 m	3	779	6.225	168.940	-3.822	130.782	-7.840	5.002	23	19	0.075	1.652	0.130	2.671	0.159	0.062	0.101	0.039	0.025	6	5	0.152	0.164	0.901	0.872	0.579	0.204	0.643	0.200	2.664
3136 m	4	882	6.244	164.549	-8.663	106.062	-11.490	5.013	24	20	0.072	1.667	0.000	2.737	0.167	0.000	-0.270	0.036	0.027	6	4	0.101	0.157	1.066	0.794	0.501	0.273	0.711	-0.535	3.609
3136 m	5	774	6.124	157.405	-6.543	111.789	-8.705	5.039	35	30	0.050	1.714	0.128	3.266	0.134	0.055	0.023	0.027	0.022	8	7	0.155	0.202	0.950	0.899	0.546	0.152	0.701	1.314	-0.145
3136 m	6	888	6.303	178.146	-5.432	133.241	-8.844	5.013	44	41	0.043	1.864	0.080	4.114	0.139	0.095	0.058	0.023	0.029	10	8	0.143	0.232	1.051	0.887	0.492	0.154	0.694	3.457	-0.910
3471 m	1	870	6.331	180.586	-4.274	127.939	-10.053	4.988	36	29	0.046	1.611	0.000	3.946	0.139	0.000	-0.014	0.023	0.033	9	7	0.097	0.248	1.018	0.871	0.513	0.177	0.738	1.125	-0.325
3471 m	2	1091	6.572	198.045	-7.543	147.273	-11.462	5.011	45	42	0.042	1.867	0.040	3.817	0.153	0.053	0.075	0.022	0.028	8	7	0.094	0.237	1.101	0.862	0.468	0.176	0.690	2.776	-0.686
3471 m	3	949	6.389	167.893	-10.145	114.811	-12.330	5.016	34	30	0.053	1.765	0.000	3.817	0.161	0.042	-0.049	0.027	0.038	9	6	0.098	0.215	1.071	0.817	0.488	0.231	0.675	2.144	1.527
3471 m	4	662	6.009	138.388	-7.244	93.307	-10.692	5.014	28	23	0.061	1.643	0.000	2.678	0.123	0.033	-0.144	0.030	0.015	6	6	0.087	0.076	0.910	0.863	0.570	0.198	0.705	-1.618	0.736
3471 m	5	635	5.923	128.881	-8.672	84.878	-10.242	5.001	20	15	0.079	1.500	0.000	2.370	0.156	0.000	-0.204	0.040	0.022	5	5	0.132	0.131	0.773	0.830	0.643	0.253	0.700	-2.454	3.510
3471 m	6	880	6.317	182.693	-4.088	137.442	-8.618	5.010	53	53	0.038	2.000	0.080	3.625	0.145	0.112	0.107	0.021	0.021	8	9	0.115	0.206	1.022	0.866	0.503	0.167	0.685	3.456	-1.728
3755 m	1	942	6.406	197.090	-2.595	159.656	-6.873	5.031	41	36	0.044	1.756	0.048	3.277	0.142	0.054	0.079	0.023	0.021	6	8	0.106	0.138	0.946	0.843	0.545	0.198	0.710	0.864	-0.523
3755 m	2	761	6.115	169.916	-2.662	125.111	-6.571	5.020	25	17	0.057	1.360	0.000	1.697	0.080	0.000	-0.124	0.027	0.003	4	8	0.068	0.015	0.566	0.945	0.734	0.130	0.830	-5.771	-1.920
3755 m	3	792	6.183	172.625	-3.235	130.764	-7.473	5.032	31	21	0.045	1.355	0.000	1.818	0.066	0.000	-0.096	0.022	0.003	4	10	0.055	0.014	0.556	0.936	0.735	0.124	0.848	-5.569	-3.284
3755 m	4	898	6.378	188.844	-3.084	140.016	-8.696	5.011	24	17	0.062	1.417	0.000	1.750	0.091	0.000	-0.259	0.030	0.004	4	7	0.069	0.016	0.643	0.943	0.699	0.272	0.810	-5.502	-0.029
3755 m	5	503	5.669	127.166	-2.617	101.275	-5.201	4.999	14	9	0.099	1.286	0.000	1.357	0.126	0.000	-0.532	0.050	0.005	2	5	0.132	0.036	0.406	0.939	0.819	0.195	0.765	-7.238	2.983
3755 m	6	848	6.176	175.896	-4.603	121.046	-8.692	5.018	21	15	0.071	1.429	0.000	1.742	0.104	0.000	-0.263	0.035	0.006	4	6	0.079	0.022	0.682	0.934	0.684	0.155	0.791	-5.293	0.100
3885 m	1	1059	6.527	205.416	-4.472	156.331	-9.157	4.998	51	46	0.036	1.804	0.037	3.384	0.123	0.044	0.072	0.019	0.016	7	9	0.104	0.172	0.998	0.868	0.515	0.166	0.746	1.382	-2.259
3885 m	2	764	6.165	170.870	-2.577	139.329	-5.770	5.017	50	49	0.040	1.960	0.087	4.099	0.157	0.104	-0.029	0.022	0.030	9	8	0.123	0.261	1.100	0.863	0.466	0.172	0.681	4.213	-0.788
3885 m	3	949	6.331	184.678	-6.051	130.214	-9.508	5.012	33	32	0.061	1.939	0.148	2.756	0.160	0.112	-0.041	0.034	0.019	6	5	0.127	0.149	1.051	0.828	0.499	0.222	0.649	1.902	1.963
3885 m	4	985	6.442	184.896	-7.030	139.216	-9.975	5.000	32	32	0.065	2.000	0.091	3.691	0.204	0.112	-0.212	0.036	0.048	9	4	0.161	0.280	1.200	0.833	0.428	0.219	0.615	4.549	3.825
3885 m	5	1097	6.552	211.412	-4.169	159.041	-8.707	5.006	34	28	0.050	1.647	0.000	3.028	0.118	0.024	-0.187	0.025	0.016	6	7	0.102	0.093	0.925	0.863	0.558	0.188	0.737	-1.125	-0.182
3885 m	6	1141	6.568	206.696	-6.694	166.151	-8.206	5.003	46	40	0.039	1.739	0.042	2.882	0.116	0.047	-0.047	0.020	0.011	6	9	0.117	0.134	0.894	0.855	0.568	0.182	0.735	0.113	-1.572
3987 m	1	1123	6.529	226.723	-1.263	171.616	-7.014	5.011	39	27	0.036	1.385	0.000	1.817	0.055	0.026	0.206	0.017	0.002	4	13	0.043	0.013	0.488	0.904	0.765	0.142	0.850	-4.881	-4.700
3987 m	2	1092	6.393	218.675	-2.248	199.175	-2.575	5.001	52	49	0.037	1.885	0.090	3.362	0.132	0.077	0.203	0.021	0.016	7	10	0.120	0.159	0.958	0.863	0.535	0.170	0.690	2.300	-2.117
3987 m	3	710	6.049	161.513	-2.837	123.028	-6.681	4.991	29	23	0.057	1.586	0.000	2.774	0.127	0.028	0.046	0.029	0.017	6	7	0.122	0.111	0.832	0.840	0.606	0.218	0.691	-1.146	0.852
3987 m	4	1012	6.317	204.423	-3.157	206.260	-0.990	5.000	38	34	0.048	1.789	0.055	2.948	0.132	0.069	0.225	0.026	0.016	6	8	0.114	0.137	0.904	0.832	0.566	0.212	0.696	0.732	-0.293
3987 m	5	998	6.434	214.202	-0.306	162.632	-7.272	4.988	49	49	0.042	2.000	0.080	3.550	0.166	0.125	0.047	0.024	0.025	8	9	0.146	0.272	0.986	0.864	0.522	0.172	0.641	3.920	-0.549
3987 m	6	924	6.084	200.318	-1.144	188.487	-1.226	4.985	39	34	0.046	1.744	0.098	2.525	0.111	0.090	0.018	0.026	0.009	6	9	0.138	0.082	0.793	0.864	0.619	0.180	0.686	-0.193	-0.875
4128 m	1	649	5.776	150.111	-3.142	103.871	-6.316	5.050	41	32	0.039	1.561	0.130	3.071	0.098	0.058	0.212	0.021	0.012	6	12	0.111	0.107	0.648	0.869	0.688	0.173	0.710	-0.668	-2.499
4128 m	2	1002	6.433	197.969	-4.397	145.487	-9.058	4.996	45	41	0.041	1.822	0.076	3.703	0.146	0.057	0.054	0.023	0.025	9	8	0.118	0.234	1.010	0.870	0.512	0.169	0.690	2.629	-0.920
4128 m	3	984	6.394	205.235	-2.009	151.426	-7.801	5.011	49	47	0.040	1.918	0.087	3.707	0.161	0.083	0.133	0.023	0.026	9	9	0.148	0.282	0.998	0.865	0.516	0.171	0.655	3.871	-0.805
4128 m	4	947	6.395	195.533	-3.180	141.545	-9.111	4.996	53	48	0.035	1.811	0.072	3.823	0.114	0.091	0.112	0.018	0.017	9	11	0.100	0.179	0.932	0.900	0.547	0.134	0.718	2.203	-3.322
4128 m	5	774	6.113	172.490	-2.584	125.307	-7.456	5.048	38	29	0.041	1.526	0.088	3.247	0.097	0.037	0.078	0.021	0.014	8	10	0.121	0.134	0.764	0.916	0.633	0.132	0.762	-0.732	-2.531
4128 m	6	1077	6.505	207.183	-4.528	149.467	-9.632	4.991	36	29	0.046	1.611	0.000	3.433	0.146	0.012	0.086	0.024	0.027	6	8	0.125	0.200	0.895	0.853	0.571	0.195	0.690	0.362	0.110

Supplementary Table 3. Pearson correlations (with *p*-values in the parentheses) among altitude, taxonomic diversity, phylogenetic diversity, co-occurrence network properties, and functional diversity. Significant correlations are shown in bold.

	**Altitude**	**Richness**	**Shannon diversity**	**Phylogenetic diversity**	**Weighted phylogenetic diversity**	**SES of phylogenetic diversity**	**SES of weighted phylogenetic diversity**	**Network structure (PC1)**	**Network structure (PC2)**	**Diversity of functional potentials**
**Richness**	**0.378(0.023)**									
**Shannon diversity**	0.267(0.116)	**0.954(<0.001)**								
**Phylogenetic diversity**	**0.498(0.002)**	**0.948(<0.001)**	**0.893(<0.001)**							
**Weighted phylogenetic diversity**	**0.501(0.002)**	**0.800(<0.001)**	**0.680(<0.001)**	**0.894(<0.001)**						
**SES of phylogenetic diversity**	**0.425(0.010)**	-0.065(0.706)	-0.140(0.415)	0.245(0.150)	**0.389(0.019)**					
**SES of weighted phylogenetic diversity**	**0.340(0.043)**	-0.114(0.506)	-0.299(0.077)	0.103(0.552)	**0.484(0.003)**	**0.717(<0.001)**				
**Network structure (PC1)**	0.018(0.917)	**0.380(0.022)**	**0.387(0.020)**	0.319(0.058)	0.285(0.092)	-0.152(0.377)	-0.135(0.432)			
**Network structure (PC2)**	**-0.547(<0.001)**	**-0.522(<0.001)**	**-0.503(0.002)**	**-0.632(<0.001)**	**-0.545(<0.001)**	-0.310(0.066)	-0.151(0.380)	0.000(1.000)		
**Diversity of functional potentials**	0.089(0.607)	-0.034(0.844)	0.018(0.915)	-0.048(0.783)	-0.144(0.403)	-0.168(0.329)	-0.138(0.421)	-0.147(0.393)	**-0.452(0.006)**	

Supplementary Table 4. Soil bacterial taxa with significantly different abundances across altitudes according to linear discriminant analysis (LDA) effect size (LEfSe). P.adj represents adjusted *p*-values obtained by controlling the false discovery rate.

Taxon	Group with higher abundance	LDA	P.adj
p__Acidobacteria|c__Acidobacteriia	3471 m	4.940	0.017
p__Acidobacteria	3471 m	4.826	0.022
p__Proteobacteria	3755 m	4.811	0.041
p__Proteobacteria|c__Gammaproteobacteria	3755 m	4.748	0.016
p__Verrucomicrobia	3471 m	4.738	0.027
p__Verrucomicrobia|c__Verrucomicrobiae	3471 m	4.738	0.027
p__Verrucomicrobia|c__Verrucomicrobiae|o__Chthoniobacterales|f__Chthoniobacteraceae|g__Candidatus_Udaeobacter	3471 m	4.726	0.025
p__Verrucomicrobia|c__Verrucomicrobiae|o__Chthoniobacterales|f__Chthoniobacteraceae	3471 m	4.724	0.027
p__Verrucomicrobia|c__Verrucomicrobiae|o__Chthoniobacterales	3471 m	4.715	0.027
p__Proteobacteria|c__Gammaproteobacteria|o__Betaproteobacteriales	3755 m	4.575	0.015
p__Acidobacteria|c__Acidobacteriia|o__Acidobacteriales	3885 m	4.562	0.024
p__Acidobacteria|c__Acidobacteriia|o__Subgroup_2	3471 m	4.548	0.021
p__Bacteroidetes|c__Bacteroidia|o__Flavobacteriales	3755 m	4.445	0.023
p__Bacteroidetes|c__Bacteroidia|o__Flavobacteriales|f__Flavobacteriaceae	3755 m	4.434	0.022
p__Bacteroidetes|c__Bacteroidia|o__Flavobacteriales|f__Flavobacteriaceae|g__Flavobacterium	3755 m	4.434	0.022
p__Acidobacteria|c__Blastocatellia_Subgroup_4	3987 m	4.403	0.043
p__Patescibacteria	3987 m	4.350	0.035
p__Acidobacteria|c__Acidobacteriia|o__Solibacterales	3471 m	4.341	0.016
p__Acidobacteria|c__Acidobacteriia|o__Solibacterales|f__Solibacteraceae_Subgroup_3	3471 m	4.341	0.016
p__Proteobacteria|c__Gammaproteobacteria|o__Betaproteobacteriales|f__Nitrosomonadaceae	3755 m	4.309	0.021
p__Nitrospirae	3755 m	4.274	0.016
p__Proteobacteria|c__Alphaproteobacteria	3755 m	4.270	0.043
p__Nitrospirae|c__Nitrospira	3755 m	4.268	0.016
p__Nitrospirae|c__Nitrospira|o__Nitrospirales	3755 m	4.268	0.016
p__Nitrospirae|c__Nitrospira|o__Nitrospirales|f__Nitrospiraceae	3755 m	4.268	0.016
p__Nitrospirae|c__Nitrospira|o__Nitrospirales|f__Nitrospiraceae|g__Nitrospira	3755 m	4.268	0.016
p__Patescibacteria|c__Parcubacteria	3987 m	4.264	0.030
p__Acidobacteria|c__Blastocatellia_Subgroup_4|o__Pyrinomonadales|f__Pyrinomonadaceae	3987 m	4.213	0.035

p__Acidobacteria|c__Blastocatellia_Subgroup_4|o__Pyrinomonadales|f__Pyrinomonadaceae|g__RB41	3987 m	4.213	0.035
p__Acidobacteria|c__Blastocatellia_Subgroup_4|o__Pyrinomonadales	3987 m	4.213	0.035
p__Acidobacteria|c__Acidobacteriia|o__Solibacterales|f__Solibacteraceae_Subgroup_3|g__Bryobacter	3471 m	4.190	0.015
p__Planctomycetes	3136 m	4.180	0.038
p__Proteobacteria|c__Gammaproteobacteria|o__Pseudomonadales|f__Moraxellaceae|g__Acinetobacter	3987 m	4.135	0.034
p__Planctomycetes|c__Phycisphaerae	3136 m	4.135	0.022
p__Proteobacteria|c__Gammaproteobacteria|o__Pseudomonadales|f__Moraxellaceae	3987 m	4.125	0.046
p__Proteobacteria|c__Gammaproteobacteria|o__Pseudomonadales|f__Moraxellaceae|g__Acinetobacter|s__Acinetobacter_calcoaceticus	3987 m	4.122	0.022
p__Firmicutes|c__Bacilli	3136 m	4.116	0.032
p__Firmicutes|c__Bacilli|o__Bacillales	3136 m	4.107	0.030
p__Proteobacteria|c__Alphaproteobacteria|o__Rhizobiales|f__Xanthobacteraceae	3471 m	4.077	0.047
p__Planctomycetes|c__Phycisphaerae|o__Tepidisphaerales|f__WD2101_soil_group	3136 m	4.068	0.018
p__Planctomycetes|c__Phycisphaerae|o__Tepidisphaerales	3136 m	4.059	0.018
p__Proteobacteria|c__Gammaproteobacteria|o__Betaproteobacteriales|f__TRA3-20	3755 m	3.950	0.042
p__Actinobacteria	4128 m	3.938	0.045
p__Acidobacteria|c__Acidobacteriia|o__Solibacterales|f__Solibacteraceae_Subgroup_3|g__Candidatus_Solibacter	3885 m	3.930	0.021
p__Acidobacteria|c__Acidobacteriia|o__Acidobacteriales|f__Acidobacteriaceae_Subgroup_1	3471 m	3.920	0.027
p__Proteobacteria|c__Alphaproteobacteria|o__Rhizobiales|f__Xanthobacteraceae|g__Bradyrhizobium	3471 m	3.884	0.015
p__Proteobacteria|c__Deltaproteobacteria	3755 m	3.879	0.046
p__Proteobacteria|c__Gammaproteobacteria|o__Betaproteobacteriales|f__Nitrosomonadaceae|g__Ellin6067	3755 m	3.860	0.016
p__Actinobacteria|c__Actinobacteria	3136 m	3.823	0.030
p__Firmicutes|c__Bacilli|o__Bacillales|f__Bacillaceae	3136 m	3.796	0.046
p__Firmicutes|c__Bacilli|o__Bacillales|f__Bacillaceae|g__Bacillus	3136 m	3.796	0.046
p__Acidobacteria|c__Acidobacteriia|o__Acidobacteriales|f__Acidobacteriaceae_Subgroup_1|g__Granulicella	3471 m	3.744	0.027
p__Proteobacteria|c__Alphaproteobacteria|o__Micropepsales	3471 m	3.733	0.022
p__Proteobacteria|c__Alphaproteobacteria|o__Micropepsales|f__Micropepsaceae	3471 m	3.733	0.022
p__Proteobacteria|c__Gammaproteobacteria|o__Betaproteobacteriales|f__Nitrosomonadaceae|g__MND1	3755 m	3.689	0.038
p__Proteobacteria|c__Gammaproteobacteria|o__Betaproteobacteriales|f__Burkholderiaceae|g__Massilia	3755 m	3.639	0.022
p__Actinobacteria|c__Acidimicrobiia|o__Actinomarinales	3755 m	3.622	0.017
p__Proteobacteria|c__Alphaproteobacteria|o__Rhizobiales|f__Rhizobiaceae	3755 m	3.587	0.046
p__Actinobacteria|c__Actinobacteria|o__Streptomycetales	3136 m	3.581	0.035
p__Actinobacteria|c__Actinobacteria|o__Streptomycetales|f__Streptomycetaceae	3136 m	3.581	0.035

p__Actinobacteria|c__Actinobacteria|o__Streptomycetales|f__Streptomycetaceae|g__Streptomyces	3136 m	3.581	0.035
p__Planctomycetes|c__Phycisphaerae|o__Phycisphaerales	3755 m	3.562	0.027
p__Planctomycetes|c__Phycisphaerae|o__Phycisphaerales|f__Phycisphaeraceae	3755 m	3.562	0.027
p__Patescibacteria|c__Parcubacteria|o__Candidatus_Kaiserbacteria	3987 m	3.552	0.015
p__Proteobacteria|c__Gammaproteobacteria|o__Betaproteobacteriales|f__Nitrosomonadaceae|g__GOUTA6	4128 m	3.550	0.017
p__Proteobacteria|c__Alphaproteobacteria|o__Caulobacterales|f__Caulobacteraceae|g__Brevundimonas	3755 m	3.545	0.038
p__Bacteroidetes|c__Bacteroidia|o__Flavobacteriales|f__Weeksellaceae	4128 m	3.535	0.027
p__Bacteroidetes|c__Bacteroidia|o__Flavobacteriales|f__Weeksellaceae|g__Chryseobacterium	4128 m	3.535	0.027
p__Verrucomicrobia|c__Verrucomicrobiae|o__Chthoniobacterales|f__Xiphinematobacteraceae	3755 m	3.501	0.015
p__Verrucomicrobia|c__Verrucomicrobiae|o__Chthoniobacterales|f__Xiphinematobacteraceae|g__Candidatus_Xiphinematobacter	3755 m	3.501	0.015
p__Planctomycetes|c__Phycisphaerae|o__Phycisphaerales|f__Phycisphaeraceae|g__AKYG587	3755 m	3.475	0.024
p__Proteobacteria|c__Gammaproteobacteria|o__Betaproteobacteriales|f__A21b	3987 m	3.432	0.018
p__Planctomycetes|c__Planctomycetacia|o__Pirellulales|f__Pirellulaceae|g__Pirellula	3755 m	3.387	0.016
p__Proteobacteria|c__Deltaproteobacteria|o__NB1-j	3755 m	3.375	0.035
p__Actinobacteria|c__Actinobacteria|o__Micrococcales|f__Micrococcaceae	4128 m	3.358	0.047
p__Proteobacteria|c__Gammaproteobacteria|o__Xanthomonadales|f__Xanthomonadaceae|g__Arenimonas	3755 m	3.315	0.029
p__Chlamydiae	3885 m	3.310	0.024
p__Chlamydiae|c__Chlamydiae	3885 m	3.310	0.025
p__Chlamydiae|c__Chlamydiae|o__Chlamydiales	3885 m	3.310	0.024
p__Acidobacteria|c__Acidobacteriia|o__Solibacterales|f__Solibacteraceae_Subgroup_3|g__Paludibaculum	3755 m	3.298	0.035
p__Proteobacteria|c__Alphaproteobacteria|o__Rhizobiales|f__|g__Bauldia	4128 m	3.297	0.022
p__Chloroflexi|c__Anaerolineae|o__RBG-13-54-9	3987 m	3.292	0.030
p__Proteobacteria|c__Gammaproteobacteria|o__Xanthomonadales|f__Rhodanobacteraceae	3885 m	3.214	0.022
p__Chloroflexi|c__Ktedonobacteria	3885 m	3.212	0.022
p__Proteobacteria|c__Alphaproteobacteria|o__Reyranellales	3136 m	3.207	0.015
p__Proteobacteria|c__Alphaproteobacteria|o__Reyranellales|f__Reyranellaceae	3136 m	3.207	0.015
p__Planctomycetes|c__Planctomycetacia|o__Pirellulales|f__Pirellulaceae|g__Pir4_lineage	3471 m	3.207	0.022
p__Proteobacteria|c__Alphaproteobacteria|o__Reyranellales|f__Reyranellaceae|g__Reyranella	3136 m	3.205	0.015
p__Bacteroidetes|c__Bacteroidia|o__Chitinophagales|f__Saprospiraceae	3755 m	3.183	0.038
p__Proteobacteria|c__Gammaproteobacteria|o__Xanthomonadales|f__Rhodanobacteraceae|g__Rhodanobacter	3885 m	3.183	0.016
p__Fusobacteria	3136 m	3.180	0.015
p__Fusobacteria|c__Fusobacteriia	3136 m	3.180	0.015

p__Fusobacteria|c__Fusobacteriia|o__Fusobacteriales	3136 m	3.180	0.015
p__Fusobacteria|c__Fusobacteriia|o__Fusobacteriales|f__Fusobacteriaceae	3136 m	3.180	0.015
p__Fusobacteria|c__Fusobacteriia|o__Fusobacteriales|f__Fusobacteriaceae|g__Cetobacterium	3136 m	3.180	0.015
p__Actinobacteria|c__Actinobacteria|o__Micrococcales|f__Micrococcaceae|g__Pseudarthrobacter	4128 m	3.174	0.049
p__Proteobacteria|c__Gammaproteobacteria|o__Enterobacteriales|f__Enterobacteriaceae|g__Plesiomonas	3136 m	3.155	0.015
p__Bacteroidetes|c__Ignavibacteria	3987 m	3.150	0.047
p__Chloroflexi|c__Ktedonobacteria|o__Ktedonobacterales	3885 m	3.136	0.025
p__Bacteroidetes|c__Ignavibacteria|o__Ignavibacteriales|f__PHOS-HE36	3755 m	3.125	0.016
p__Actinobacteria|c__Actinobacteria|o__Frankiales	3471 m	3.124	0.022
p__Chloroflexi|c__Ktedonobacteria|o__Ktedonobacterales|f__Ktedonobacteraceae	3885 m	3.123	0.034
p__Verrucomicrobia|c__Verrucomicrobiae|o__Pedosphaerales|f__Pedosphaeraceae|g__ADurbBin063-1	3471 m	3.108	0.050
p__Proteobacteria|c__Gammaproteobacteria|o__Betaproteobacteriales|f__Rhodocyclaceae	3987 m	3.088	0.022
p__Bacteroidetes|c__Bacteroidia|o__Sphingobacteriales|f__Sphingobacteriaceae|g__Solitalea	3755 m	3.086	0.022
p__Proteobacteria|c__Alphaproteobacteria|o__Elsterales	3471 m	3.084	0.038
p__Acidobacteria|c__Acidobacteriia|o__Acidobacteriales|f__Koribacteraceae|g__Candidatus_Koribacter	3471 m	3.079	0.032
p__Acidobacteria|c__Acidobacteriia|o__Acidobacteriales|f__Koribacteraceae	3471 m	3.079	0.032
p__Proteobacteria|c__Gammaproteobacteria|o__Betaproteobacteriales|f__Burkholderiaceae|g__Rivibacter	3755 m	3.078	0.015
p__Proteobacteria|c__Alphaproteobacteria|o__Rhizobiales|f__Beijerinckiaceae|g__Roseiarcus	3471 m	3.062	0.015
p__Patescibacteria|c__WS6_Dojkabacteria	3987 m	3.030	0.023
p__Proteobacteria|c__Gammaproteobacteria|o__Betaproteobacteriales|f__Burkholderiaceae|g__Ramlibacter	3755 m	3.027	0.002
p__Proteobacteria|c__Alphaproteobacteria|o__Rhizobiales|f__Amb-16S-1323	3885 m	3.024	0.048
p__Acidobacteria|c__Subgroup_25	3885 m	3.011	0.038
p__Bacteroidetes|c__Bacteroidia|o__Cytophagales|f__Hymenobacteraceae	3755 m	3.001	0.029
p__Firmicutes|c__Clostridia|o__Clostridiales|f__Peptostreptococcaceae	4128 m	2.990	0.027
p__Actinobacteria|c__Actinobacteria|o__Frankiales|f__Acidothermaceae	3471 m	2.990	0.015
p__Actinobacteria|c__Actinobacteria|o__Frankiales|f__Acidothermaceae|g__Acidothermus	3471 m	2.990	0.015
p__Chloroflexi|c__Ktedonobacteria|o__Ktedonobacterales|f__Ktedonobacteraceae|g__HSB_OF53-F07	3885 m	2.983	0.015
p__Bacteroidetes|c__Bacteroidia|o__Cytophagales|f__Hymenobacteraceae|g__Adhaeribacter	3755 m	2.981	0.027
p__Chloroflexi|c__AD3	4128 m	2.962	0.016
p__Bacteroidetes|c__Ignavibacteria|o__Kryptoniales|f__BSV26	3987 m	2.958	0.022
p__Bacteroidetes|c__Ignavibacteria|o__Kryptoniales	3987 m	2.958	0.024
p__Proteobacteria|c__Gammaproteobacteria|o__Pseudomonadales|f__Moraxellaceae|g__Alkanindiges	4128 m	2.958	0.027

p__Acidobacteria|c__Acidobacteriia|o__Acidobacteriales|f__Acidobacteriaceae_Subgroup_1|g__Edaphobacter	3471 m	2.954	0.022
p__Patescibacteria|c__Parcubacteria|o__Candidatus_Adlerbacteria	3987 m	2.915	0.027
p__Proteobacteria|c__Gammaproteobacteria|o__Enterobacteriales|f__Enterobacteriaceae|g__Rosenbergiella	3755 m	2.914	0.027
p__Firmicutes|c__Clostridia|o__Clostridiales|f__Peptostreptococcaceae|g__Romboutsia	4128 m	2.897	0.022
p__Chloroflexi|c__Ktedonobacteria|o__C0119	3885 m	2.892	0.038
p__Proteobacteria|c__Gammaproteobacteria|o__JG36-GS-52	3987 m	2.889	0.015
p__Planctomycetes|c__Planctomycetacia|o__Pirellulales|f__Pirellulaceae|g__Blastopirellula	3755 m	2.885	0.022
p__Planctomycetes|c__Phycisphaerae|o__CCM11a	3755 m	2.883	0.043
p__Chlamydiae|c__Chlamydiae|o__Chlamydiales|f__Parachlamydiaceae	3987 m	2.876	0.035
p__Proteobacteria|c__Gammaproteobacteria|o__JG36-TzT-191	3885 m	2.870	0.038
p__Proteobacteria|c__Gammaproteobacteria|o__Betaproteobacteriales|f__Rhodocyclaceae|g__Candidatus_Accumulibacter	3987 m	2.844	0.022
p__Latescibacteria|c__Latescibacteria	3755 m	2.829	0.048
p__Proteobacteria|c__Deltaproteobacteria|o__Bdellovibrionales|f__Bdellovibrionaceae|g__Bdellovibrio|s__Bdellovibrio_sp_MPA	3755 m	2.812	0.017
p__Patescibacteria|c__Parcubacteria|o__Candidatus_Campbellbacteria	3755 m	2.812	0.027
p__Proteobacteria|c__Gammaproteobacteria|o__Diplorickettsiales|f__Diplorickettsiaceae|g__Aquicella	3136 m	2.807	0.027
p__Proteobacteria|c__Alphaproteobacteria|o__Rhodobacterales	3755 m	2.775	0.047
p__Proteobacteria|c__Alphaproteobacteria|o__Rhodobacterales|f__Rhodobacteraceae	3755 m	2.775	0.046
p__Bacteroidetes|c__Bacteroidia|o__Cytophagales|f__Cytophagaceae|g__Sporocytophaga	3755 m	2.755	0.030
p__Proteobacteria|c__Alphaproteobacteria|o__Acetobacterales|f__Acetobacteraceae|g__Acidocella	3471 m	2.752	0.030
p__Planctomycetes|c__Phycisphaerae|o__Phycisphaerales|f__Phycisphaeraceae|g__Urania-1B-19_marine_sediment_group	3755 m	2.750	0.016
p__Proteobacteria|c__Gammaproteobacteria|o__Betaproteobacteriales|f__Burkholderiaceae|g__Hydrogenophaga	3755 m	2.737	0.016
p__Bacteroidetes|c__Bacteroidia|o__Bacteroidales|f__Bacteroidetes_vadinHA17	3987 m	2.735	0.047
p__Proteobacteria|c__Gammaproteobacteria|o__Diplorickettsiales	3136 m	2.695	0.026
p__Proteobacteria|c__Gammaproteobacteria|o__Diplorickettsiales|f__Diplorickettsiaceae	3136 m	2.695	0.030
p__Proteobacteria|c__Deltaproteobacteria|o__Myxococcales|f__Polyangiaceae|g__Polyangium	3755 m	2.689	0.046
p__Chlamydiae|c__Chlamydiae|o__Chlamydiales|f__Parachlamydiaceae|g__Neochlamydia	3987 m	2.681	0.030
p__Proteobacteria|c__Alphaproteobacteria|o__Azospirillales|f__Azospirillaceae	3755 m	2.677	0.046
p__Proteobacteria|c__Alphaproteobacteria|o__Azospirillales|f__Azospirillaceae|g__Skermanella	3755 m	2.677	0.046
p__Proteobacteria|c__Gammaproteobacteria|o__Pseudomonadales|f__Pseudomonadaceae|g__Pseudomonas|s__Pseudomonas_peli	3755 m	2.640	0.046
p__Firmicutes|c__Erysipelotrichia	4128 m	2.340	0.046
p__Firmicutes|c__Erysipelotrichia|o__Erysipelotrichales	4128 m	2.340	0.046
p__Firmicutes|c__Erysipelotrichia|o__Erysipelotrichales|f__Erysipelotrichaceae	4128 m	2.340	0.046

p__Firmicutes|c__Erysipelotrichia|o__Erysipelotrichales|f__Erysipelotrichaceae|g__Turicibacter	4128 m	2.340	0.046

Supplementary Table 5. Soil bacterial functional potentials with significantly different abundances across altitudes according to linear discriminant analysis (LDA) effect size (LEfSe). P.adj represents adjusted *p*-values obtained by controlling the false discovery rate.

Pathway	Group with higher abundance	LDA	P.adj
L1__Metabolism|L2__Chemical structure transformation maps|L3__Biosynthesis of terpenoids and steroids	3471 m	3.469	0.026
L1__Metabolism|L2__Chemical structure transformation maps	3471 m	3.469	0.026
L1__Metabolism	3471 m	3.404	0.019
L1__Human Diseases	3755 m	3.190	0.029
L1__Metabolism|L2__Carbohydrate metabolism	3136 m	3.031	0.017
L1__Metabolism|L2__Amino acid metabolism	3755 m	3.012	0.046
L1__Metabolism|L2__Glycan biosynthesis and metabolism	3136 m	3.005	0.045
L1__Metabolism|L2__Metabolism of other amino acids|L3__D-Glutamine and D-glutamate metabolism	3987 m	2.842	0.020
L1__Metabolism|L2__Energy metabolism	3987 m	2.818	0.043
L1__Environmental Information Processing	3755 m	2.809	0.047
L1__Metabolism|L2__Metabolism of terpenoids and polyketides|L3__Limonene and pinene degradation	3755 m	2.778	0.041
L1__Metabolism|L2__Biosynthesis of other secondary metabolites|L3__Prodigiosin biosynthesis	3885 m	2.670	0.027
L1__Metabolism|L2__Amino acid metabolism|L3__Valine, leucine and isoleucine degradation	3755 m	2.596	0.048
L1__Metabolism|L2__Metabolism of other amino acids	3136 m	2.586	0.042
L1__Genetic Information Processing|L2__Replication and repair|L3__Mismatch repair	3987 m	2.571	0.045
L1__Metabolism|L2__Xenobiotics biodegradation and metabolism|L3__Furfural degradation	3471 m	2.571	0.046
L1__Metabolism|L2__Glycan biosynthesis and metabolism|L3__Peptidoglycan biosynthesis	3136 m	2.566	0.031
L1__Cellular Processes|L2__Cellular community - prokaryotes|L3__Biofilm formation - Pseudomonas aeruginosa	3755 m	2.557	0.026
L1__Organismal Systems|L2__Excretory system|L3__Proximal tubule bicarbonate reclamation	3471 m	2.546	0.017
L1__Organismal Systems|L2__Excretory system	3471 m	2.546	0.017
L1__Human Diseases|L2__Drug resistance: antineoplastic	3755 m	2.528	0.019
L1__Human Diseases|L2__Neurodegenerative disease	3755 m	2.506	0.042
L1__Genetic Information Processing|L2__Replication and repair|L3__Homologous recombination	3987 m	2.495	0.039
L1__Metabolism|L2__Energy metabolism|L3__Nitrogen metabolism	3755 m	2.491	0.017
L1__Metabolism|L2__Amino acid metabolism|L3__Tryptophan metabolism	3755 m	2.482	0.042
L1__Metabolism|L2__Nucleotide metabolism	3136 m	2.477	0.045
L1__Environmental Information Processing|L2__Membrane transport|L3__Bacterial secretion system	3755 m	2.472	0.014
L1__Metabolism|L2__Xenobiotics biodegradation and metabolism|L3__Drug metabolism - other enzymes	3755 m	2.465	0.026

L1__Human Diseases|L2__Cancer: overview|L3__Chemical carcinogenesis	3755 m	2.463	0.046
L1__Environmental Information Processing|L2__Signal transduction	4128 m	2.456	0.020
L1__Metabolism|L2__Carbohydrate metabolism|L3__Ascorbate and aldarate metabolism	3471 m	2.455	0.042
L1__Metabolism|L2__Xenobiotics biodegradation and metabolism|L3__Chloroalkane and chloroalkene degradation	3471 m	2.453	0.043
L1__Metabolism|L2__Xenobiotics biodegradation and metabolism|L3__Nitrotoluene degradation	3755 m	2.448	0.014
L1__Human Diseases|L2__Cancer: overview	3755 m	2.445	0.042
L1__Genetic Information Processing|L2__Replication and repair|L3__Non-homologous end-joining	3471 m	2.439	0.014
L1__Human Diseases|L2__Drug resistance: antimicrobial|L3__Vancomycin resistance	3136 m	2.425	0.046
L1__Metabolism|L2__Biosynthesis of other secondary metabolites|L3__Betalain biosynthesis	3755 m	2.421	0.020
L1__Metabolism|L2__Glycan biosynthesis and metabolism|L3__Glycosphingolipid biosynthesis - globo and isoglobo series	3885 m	2.420	0.039
L1__Metabolism|L2__Carbohydrate metabolism|L3__Galactose metabolism	3885 m	2.406	0.014
L1__Human Diseases|L2__Drug resistance: antineoplastic|L3__Platinum drug resistance	3755 m	2.403	0.029
L1__Metabolism|L2__Carbohydrate metabolism|L3__Pentose phosphate pathway	3471 m	2.395	0.014
L1__Metabolism|L2__Metabolism of other amino acids|L3__D-Arginine and D-ornithine metabolism	3755 m	2.368	0.017
L1__Metabolism|L2__Xenobiotics biodegradation and metabolism|L3__Chlorocyclohexane and chlorobenzene degradation	3471 m	2.366	0.024
L1__Metabolism|L2__Metabolism of terpenoids and polyketides|L3__Insect hormone biosynthesis	3471 m	2.356	0.026
L1__Metabolism|L2__Carbohydrate metabolism|L3__Starch and sucrose metabolism	3136 m	2.355	0.017
L1__Cellular Processes|L2__Cellular community - prokaryotes|L3__Biofilm formation - Vibrio cholerae	3755 m	2.316	0.014
L1__Metabolism|L2__Metabolism of other amino acids|L3__Selenocompound metabolism	3471 m	2.309	0.040
L1__Metabolism|L2__Metabolism of other amino acids|L3__Taurine and hypotaurine metabolism	3471 m	2.301	0.017
L1__Metabolism|L2__Lipid metabolism|L3__Biosynthesis of unsaturated fatty acids	3471 m	2.300	0.050
L1__Metabolism|L2__Amino acid metabolism|L3__Arginine biosynthesis	3987 m	2.294	0.027
L1__Metabolism|L2__Carbohydrate metabolism|L3__Propanoate metabolism	3755 m	2.293	0.017
L1__Metabolism|L2__Carbohydrate metabolism|L3__Glyoxylate and dicarboxylate metabolism	4128 m	2.289	0.017
L1__Metabolism|L2__Carbohydrate metabolism|L3__Pentose and glucuronate interconversions	3471 m	2.281	0.014
L1__Metabolism|L2__Amino acid metabolism|L3__Lysine biosynthesis	3987 m	2.270	0.040
L1__Metabolism|L2__Amino acid metabolism|L3__Lysine degradation	3755 m	2.267	0.041
L1__Metabolism|L2__Carbohydrate metabolism|L3__Amino sugar and nucleotide sugar metabolism	3136 m	2.267	0.018
L1__Environmental Information Processing|L2__Signal transduction|L3__Two-component system	3755 m	2.265	0.047
L1__Cellular Processes|L2__Cellular community - prokaryotes|L3__Quorum sensing	3471 m	2.264	0.039
L1__Human Diseases|L2__Cardiovascular disease	3755 m	2.261	0.026
L1__Metabolism|L2__Amino acid metabolism|L3__Cysteine and methionine metabolism	3755 m	2.258	0.024

L1__Human Diseases|L2__Cancer: specific types	3755 m	2.247	0.026
L1__Metabolism|L2__Lipid metabolism|L3__Ether lipid metabolism	3471 m	2.240	0.024
L1__Genetic Information Processing|L2__Replication and repair|L3__Base excision repair	3885 m	2.239	0.041
L1__Organismal Systems|L2__Endocrine system	3987 m	2.197	0.043
L1__Metabolism|L2__Amino acid metabolism|L3__Histidine metabolism	3755 m	2.173	0.021
L1__Metabolism|L2__Carbohydrate metabolism|L3__Fructose and mannose metabolism	3136 m	2.160	0.017
L1__Metabolism|L2__Biosynthesis of other secondary metabolites|L3__Monobactam biosynthesis	3885 m	2.154	0.029
L1__Human Diseases|L2__Infectious disease: bacterial	3755 m	2.144	0.044
L1__Metabolism|L2__Global and overview maps|L3__Fatty acid metabolism	3755 m	2.144	0.030
L1__Metabolism|L2__Glycan biosynthesis and metabolism|L3__Glycosaminoglycan degradation	3885 m	2.136	0.047
L1__Metabolism|L2__Metabolism of cofactors and vitamins|L3__Ubiquinone and other terpenoid-quinone biosynthesis	3885 m	2.114	0.044
L1__Metabolism|L2__Nucleotide metabolism|L3__Purine metabolism	3987 m	2.105	0.039
L1__Metabolism|L2__Metabolism of cofactors and vitamins|L3__Porphyrin and chlorophyll metabolism	3755 m	2.101	0.019
L1__Metabolism|L2__Lipid metabolism|L3__alpha-Linolenic acid metabolism	3755 m	2.090	0.026
L1__Environmental Information Processing|L2__Membrane transport|L3__Phosphotransferase system (PTS)	3136 m	2.081	0.042
L1__Metabolism|L2__Xenobiotics biodegradation and metabolism|L3__Ethylbenzene degradation	3755 m	2.072	0.044
L1__Human Diseases|L2__Cardiovascular disease|L3__Fluid shear stress and atherosclerosis	3755 m	2.072	0.020
L1__Metabolism|L2__Metabolism of terpenoids and polyketides|L3__Biosynthesis of siderophore group nonribosomal peptides	3987 m	2.071	0.046
L1__Metabolism|L2__Carbohydrate metabolism|L3__Pyruvate metabolism	3987 m	2.070	0.042
L1__Metabolism|L2__Lipid metabolism|L3__Linoleic acid metabolism	3755 m	2.062	0.033
L1__Metabolism|L2__Metabolism of cofactors and vitamins|L3__Riboflavin metabolism	3987 m	2.037	0.040
L1__Organismal Systems|L2__Aging|L3__Longevity regulating pathway - worm	3987 m	2.032	0.043
L1__Metabolism|L2__Energy metabolism|L3__Methane metabolism	3755 m	2.017	0.024
L1__Metabolism|L2__Lipid metabolism|L3__Glycerophospholipid metabolism	3987 m	2.017	0.024
L1__Metabolism|L2__Carbohydrate metabolism|L3__Inositol phosphate metabolism	3471 m	2.009	0.043
L1__Human Diseases|L2__Infectious disease: viral	3755 m	2.002	0.039
L1__Organismal Systems|L2__Circulatory system|L3__Cardiac muscle contraction	3755 m	1.976	0.017
L1__Organismal Systems|L2__Circulatory system	3755 m	1.974	0.017
L1__Metabolism|L2__Biosynthesis of other secondary metabolites|L3__Neomycin, kanamycin and gentamicin biosynthesis	3885 m	1.973	0.017
L1__Metabolism|L2__Glycan biosynthesis and metabolism|L3__Lipoarabinomannan (LAM) biosynthesis	3471 m	1.969	0.043
L1__Cellular Processes|L2__Cell growth and death|L3__Ferroptosis	3136 m	1.963	0.022
L1__Human Diseases|L2__Cancer: specific types|L3__Hepatocellular carcinoma	3755 m	1.943	0.040

L1__Metabolism|L2__Metabolism of cofactors and vitamins|L3__Retinol metabolism	3471 m	1.942	0.032
L1__Metabolism|L2__Biosynthesis of other secondary metabolites|L3__Biosynthesis of various secondary metabolites - part 1	3755 m	1.936	0.017
L1__Human Diseases|L2__Infectious disease: parasitic	3755 m	1.902	0.024
L1__Organismal Systems|L2__Endocrine system|L3__PPAR signaling pathway	3471 m	1.899	0.048
L1__Human Diseases|L2__Cardiovascular disease|L3__Viral myocarditis	3755 m	1.880	0.043
L1__Organismal Systems|L2__Endocrine system|L3__Thyroid hormone signaling pathway	3471 m	1.875	0.017
L1__Cellular Processes|L2__Cell growth and death|L3__Apoptosis - multiple species	3755 m	1.869	0.043
L1__Human Diseases|L2__Endocrine and metabolic disease|L3__Insulin resistance	3987 m	1.854	0.039
L1__Human Diseases|L2__Neurodegenerative disease|L3__Parkinson disease	3755 m	1.850	0.019
L1__Human Diseases|L2__Neurodegenerative disease|L3__Alzheimer disease	3755 m	1.850	0.017
L1__Metabolism|L2__Lipid metabolism|L3__Glycerolipid metabolism	3471 m	1.850	0.033
L1__Human Diseases|L2__Endocrine and metabolic disease|L3__Non-alcoholic fatty liver disease (NAFLD)	3755 m	1.831	0.018
L1__Metabolism|L2__Metabolism of terpenoids and polyketides|L3__Sesquiterpenoid and triterpenoid biosynthesis	3471 m	1.830	0.025
L1__Organismal Systems|L2__Environmental adaptation	3755 m	1.821	0.020
L1__Metabolism|L2__Biosynthesis of other secondary metabolites|L3__Glucosinolate biosynthesis	3987 m	1.804	0.043
L1__Metabolism|L2__Biosynthesis of other secondary metabolites|L3__Staurosporine biosynthesis	3755 m	1.795	0.047
L1__Human Diseases|L2__Neurodegenerative disease|L3__Huntington disease	3755 m	1.793	0.023
L1__Metabolism|L2__Metabolism of terpenoids and polyketides|L3__Carotenoid biosynthesis	3471 m	1.770	0.026
L1__Organismal Systems|L2__Immune system	3755 m	1.735	0.042
L1__Metabolism|L2__Biosynthesis of other secondary metabolites|L3__Indole alkaloid biosynthesis	3987 m	1.726	0.042
L1__Organismal Systems|L2__Environmental adaptation|L3__Thermogenesis	3755 m	1.725	0.017
L1__Environmental Information Processing|L2__Signal transduction|L3__MAPK signaling pathway - plant	3987 m	1.720	0.039
L1__Human Diseases|L2__Infectious disease: parasitic|L3__African trypanosomiasis	3755 m	1.705	0.020
L1__Genetic Information Processing|L2__Translation|L3__RNA transport	3755 m	1.704	0.017
L1__Cellular Processes|L2__Cell growth and death|L3__Apoptosis - fly	3755 m	1.672	0.046
L1__Organismal Systems|L2__Environmental adaptation|L3__Plant-pathogen interaction	3885 m	1.666	0.038
L1__Metabolism|L2__Biosynthesis of other secondary metabolites|L3__Stilbenoid, diarylheptanoid and gingerol biosynthesis	3755 m	1.665	0.017
L1__Cellular Processes|L2__Cell growth and death|L3__Necroptosis	3885 m	1.654	0.019
L1__Cellular Processes|L2__Cell growth and death|L3__p53 signaling pathway	3755 m	1.644	0.043
L1__Human Diseases|L2__Cancer: specific types|L3__Colorectal cancer	3755 m	1.613	0.043
L1__Metabolism|L2__Metabolism of terpenoids and polyketides|L3__Biosynthesis of type II polyketide backbone	3136 m	1.605	0.022
L1__Human Diseases|L2__Cancer: specific types|L3__Small cell lung cancer	3755 m	1.599	0.042

L1__Genetic Information Processing|L2__Folding, sorting and degradation|L3__Proteasome	3755 m	1.591	0.017
L1__Organismal Systems|L2__Endocrine system|L3__Adipocytokine signaling pathway	3136 m	1.581	0.044
L1__Metabolism|L2__Biosynthesis of other secondary metabolites|L3__Isoflavonoid biosynthesis	3136 m	1.552	0.033
L1__Cellular Processes|L2__Cell growth and death|L3__Apoptosis	3755 m	1.534	0.040
L1__Human Diseases|L2__Cancer: overview|L3__Pathways in cancer	3755 m	1.525	0.035
L1__Human Diseases|L2__Infectious disease: parasitic|L3__Toxoplasmosis	3755 m	1.497	0.045
L1__Cellular Processes|L2__Cell growth and death|L3__Meiosis - yeast	3471 m	1.495	0.024
L1__Genetic Information Processing|L2__Translation|L3__Ribosome biogenesis in eukaryotes	3755 m	1.478	0.014
L1__Organismal Systems|L2__Immune system|L3__NOD-like receptor signaling pathway	3755 m	1.475	0.017
L1__Metabolism|L2__Biosynthesis of other secondary metabolites|L3__Flavonoid biosynthesis	3755 m	1.429	0.017
L1__Human Diseases|L2__Infectious disease: viral|L3__Hepatitis B	3755 m	1.418	0.043
L1__Organismal Systems|L2__Immune system|L3__Antigen processing and presentation	3987 m	1.393	0.033
L1__Human Diseases|L2__Infectious disease: viral|L3__Influenza A	3755 m	1.382	0.043
L1__Metabolism|L2__Biosynthesis of other secondary metabolites|L3__Caffeine metabolism	3471 m	1.380	0.024
L1__Human Diseases|L2__Cancer: specific types|L3__Renal cell carcinoma	3755 m	1.355	0.017
L1__Human Diseases|L2__Infectious disease: viral|L3__Herpes simplex virus 1 infection	3755 m	1.309	0.043
L1__Cellular Processes|L2__Transport and catabolism|L3__Autophagy - yeast	3755 m	1.288	0.048
L1__Human Diseases|L2__Infectious disease: viral|L3__Kaposi sarcoma-associated herpesvirus infection	3755 m	1.274	0.042
L1__Organismal Systems|L2__Endocrine system|L3__Progesterone-mediated oocyte maturation	3987 m	1.211	0.031
L1__Human Diseases|L2__Infectious disease: viral|L3__Human cytomegalovirus infection	3755 m	1.203	0.043
L1__Genetic Information Processing|L2__Transcription|L3__Basal transcription factors	3471 m	1.143	0.026
L1__Organismal Systems|L2__Endocrine system|L3__Estrogen signaling pathway	3987 m	1.133	0.035
L1__Human Diseases|L2__Cancer: overview|L3__MicroRNAs in cancer	3755 m	1.128	0.026
L1__Human Diseases|L2__Infectious disease: parasitic|L3__Amoebiasis	3136 m	1.128	0.029
L1__Human Diseases|L2__Cancer: specific types|L3__Prostate cancer	3987 m	1.126	0.031
L1__Organismal Systems|L2__Immune system|L3__IL-17 signaling pathway	3987 m	1.118	0.028
L1__Organismal Systems|L2__Immune system|L3__Th17 cell differentiation	3987 m	1.097	0.030
L1__Organismal Systems|L2__Immune system|L3__RIG-I-like receptor signaling pathway	3471 m	1.085	0.024
L1__Human Diseases|L2__Endocrine and metabolic disease|L3__Cushing syndrome	3755 m	1.043	0.017
L1__Human Diseases|L2__Infectious disease: bacterial|L3__Vibrio cholerae infection	3755 m	0.908	0.035
L1__Environmental Information Processing|L2__Signal transduction|L3__PI3K-Akt signaling pathway	3987 m	0.891	0.040
L1__Human Diseases|L2__Infectious disease: bacterial|L3__Bacterial invasion of epithelial cells	4128 m	0.881	0.026

L1__Environmental Information Processing|L2__Signal transduction|L3__TNF signaling pathway	3755 m	0.832	0.021
L1__Environmental Information Processing|L2__Signal transduction|L3__Notch signaling pathway	4128 m	0.826	0.019
L1__Organismal Systems|L2__Nervous system|L3__Retrograde endocannabinoid signaling	3755 m	0.787	0.017
L1__Cellular Processes|L2__Cellular community - eukaryotes	3136 m	0.714	0.029
L1__Environmental Information Processing|L2__Signal transduction|L3__cAMP signaling pathway	3755 m	0.689	0.019
L1__Human Diseases|L2__Infectious disease: bacterial|L3__Pathogenic Escherichia coli infection	4128 m	0.637	0.036
L1__Organismal Systems|L2__Endocrine system|L3__Parathyroid hormone synthesis, secretion and action	3755 m	0.613	0.024
L1__Organismal Systems|L2__Endocrine system|L3__Ovarian steroidogenesis	3755 m	0.613	0.017
L1__Environmental Information Processing|L2__Signal transduction|L3__Ras signaling pathway	3885 m	0.611	0.014
L1__Human Diseases|L2__Cancer: specific types|L3__Chronic myeloid leukemia	4128 m	0.588	0.019
L1__Cellular Processes|L2__Cell growth and death|L3__Cellular senescence	3471 m	0.586	0.014
L1__Organismal Systems|L2__Circulatory system|L3__Vascular smooth muscle contraction	3471 m	0.581	0.017
L1__Cellular Processes|L2__Cellular community - eukaryotes|L3__Gap junction	3136 m	0.549	0.017
L1__Organismal Systems|L2__Immune system|L3__Chemokine signaling pathway	3755 m	0.542	0.039
L1__Environmental Information Processing|L2__Signal transduction|L3__NF-kappa B signaling pathway	3755 m	0.539	0.027
L1__Environmental Information Processing|L2__Signal transduction|L3__Apelin signaling pathway	3471 m	0.520	0.024
L1__Organismal Systems|L2__Digestive system|L3__Salivary secretion	3471 m	0.465	0.017
L1__Human Diseases|L2__Infectious disease: parasitic|L3__Leishmaniasis	3755 m	0.459	0.027
L1__Cellular Processes|L2__Transport and catabolism|L3__Phagosome	3136 m	0.436	0.024
L1__Organismal Systems|L2__Immune system|L3__Fc gamma R-mediated phagocytosis	3755 m	0.433	0.039
L1__Organismal Systems|L2__Endocrine system|L3__Regulation of lipolysis in adipocytes	3755 m	0.431	0.027
L1__Environmental Information Processing|L2__Signal transduction|L3__Wnt signaling pathway	4128 m	0.430	0.022
L1__Cellular Processes|L2__Cellular community - eukaryotes|L3__Adherens junction	3755 m	0.429	0.039
L1__Cellular Processes|L2__Transport and catabolism|L3__Endocytosis	4128 m	0.426	0.021
L1__Environmental Information Processing|L2__Signal transduction|L3__VEGF signaling pathway	3755 m	0.411	0.027
L1__Cellular Processes|L2__Cellular community - eukaryotes|L3__Tight junction	3136 m	0.405	0.024
L1__Organismal Systems|L2__Endocrine system|L3__GnRH signaling pathway	3885 m	0.395	0.021
L1__Organismal Systems|L2__Sensory system|L3__Phototransduction	3471 m	0.391	0.021
L1__Organismal Systems|L2__Nervous system|L3__Cholinergic synapse	3136 m	0.364	0.021

Supplementary Table 6. Comparison of candidate models for explaining the diversity of soil bacterial functional potentials. Significant variables are shown in bold.

Variable	Estimate	Std. Error	*t* value	*p*-value
**Original model: R^2_{adj}=0.346; AICc=96.4**				
Shannon diversity	0.030	0.202	0.146	0.885
**Weighted phylogenetic diversity**	**-0.574**	**0.198**	**-2.895**	**0.007**
Network structure (PC1)	0.005	0.154	0.035	0.973
**Network structure (PC2)**	**-0.750**	**0.173**	**-4.325**	**0.000**
**Model without PC2: R^2_{adj}=-0.016; AICc=110.5**				
Shannon diversity	0.279	0.242	1.153	0.257
Weighted phylogenetic diversity	-0.284	0.233	-1.221	0.231
Network structure (PC1)	-0.174	0.185	-0.940	0.354
**Model without phylogenetic diversity: R^2_{adj}=0.195; AICc=102.1**				
Shannon diversity	-0.255	0.196	-1.297	0.204
Network structure (PC1)	-0.048	0.170	-0.284	0.779
**Network structure (PC2)**	**-0.580**	**0.181**	**-3.205**	**0.003**
**Updated model: R^2_{adj}=0.422; AICc=93.8**				
Shannon diversity	-0.146	0.206	-0.712	0.482
**Weighted phylogenetic diversity**	**-0.453**	**0.194**	**-2.336**	**0.026**
Network structure (PC1)	-0.022	0.145	-0.151	0.881
**Network structure (PC2)**	**-0.548**	**0.186**	**-2.949**	**0.006**
**Weighted phylogenetic diversity:PC2**	**0.281**	**0.124**	**2.256**	**0.032**

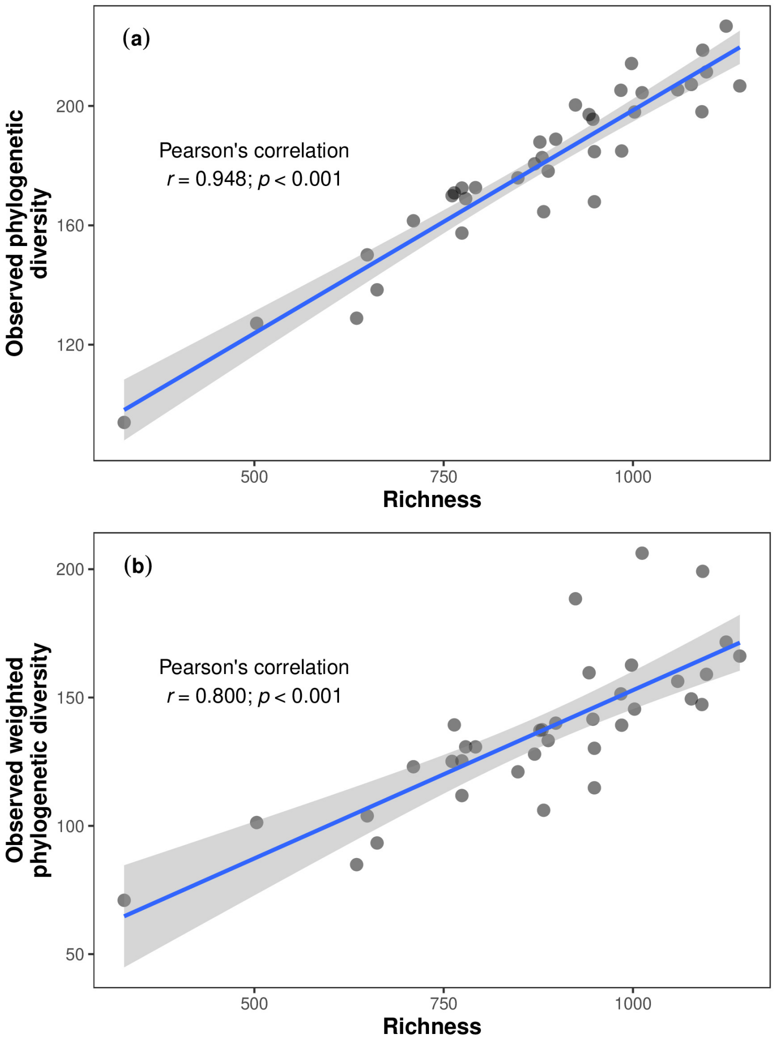

Supplementary Figure 1. Scatter plots depicting the relationships between bacterial richness and observed phylogenetic diversity (unweighted Faith's index) (a) and observed weighted phylogenetic diversity (weighted Faith's index) (b), respectively. The inset text displays Pearson's correlation coefficient and *p*-value.

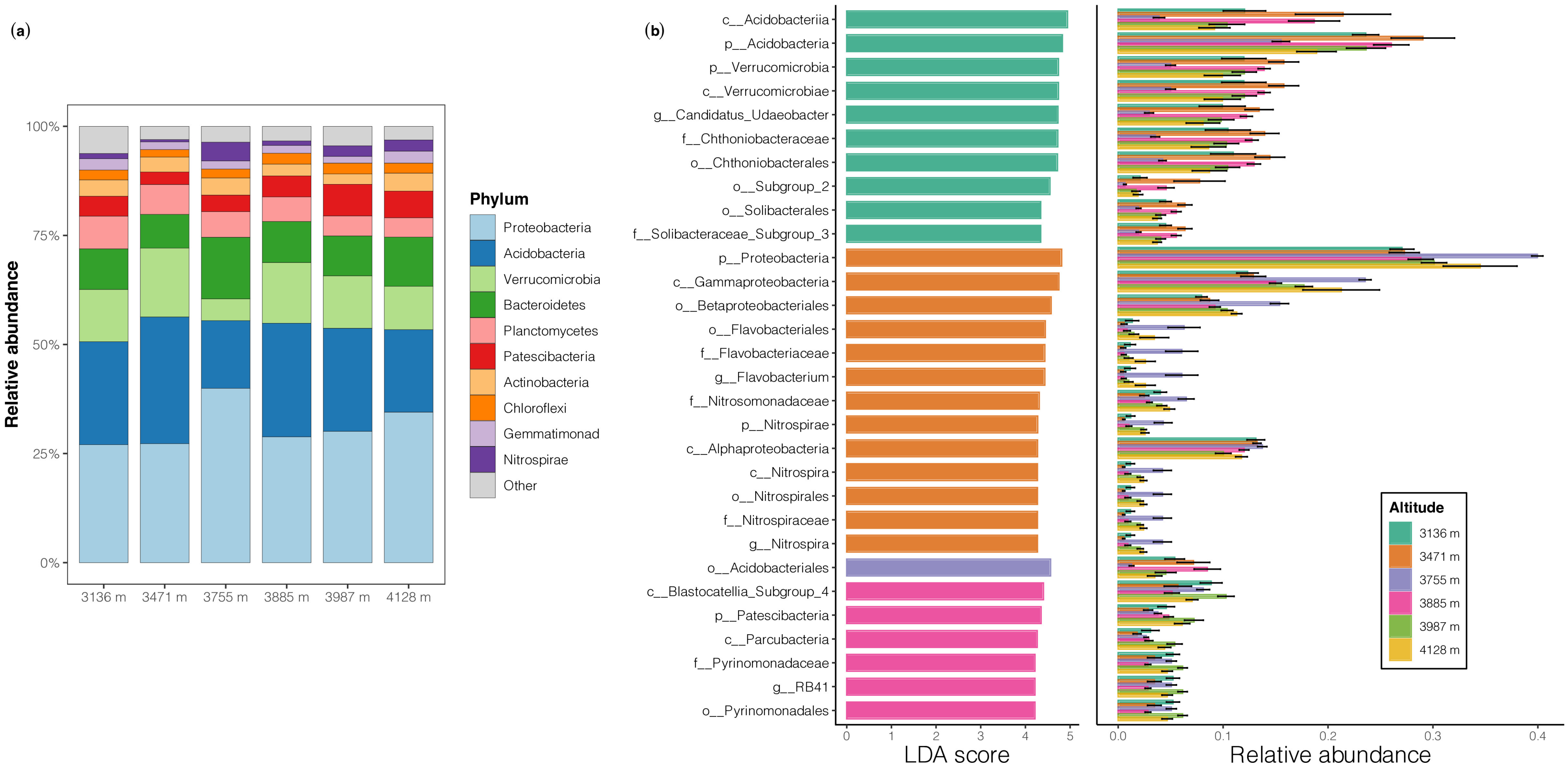

Supplementary Figure 2. Soil bacterial abundance and differences across altitudes. (a) Relative abundance of soil bacteria at the phylum level across altitudes. (b) Soil bacterial taxa that are differentially abundant across altitudes. The taxa were identified using linear discriminant analysis effect size (LEfSe). Only the top 30 taxa are shown in the figure; please refer to Supplementary Table 4 for all taxa with significantly different abundances across altitudes.

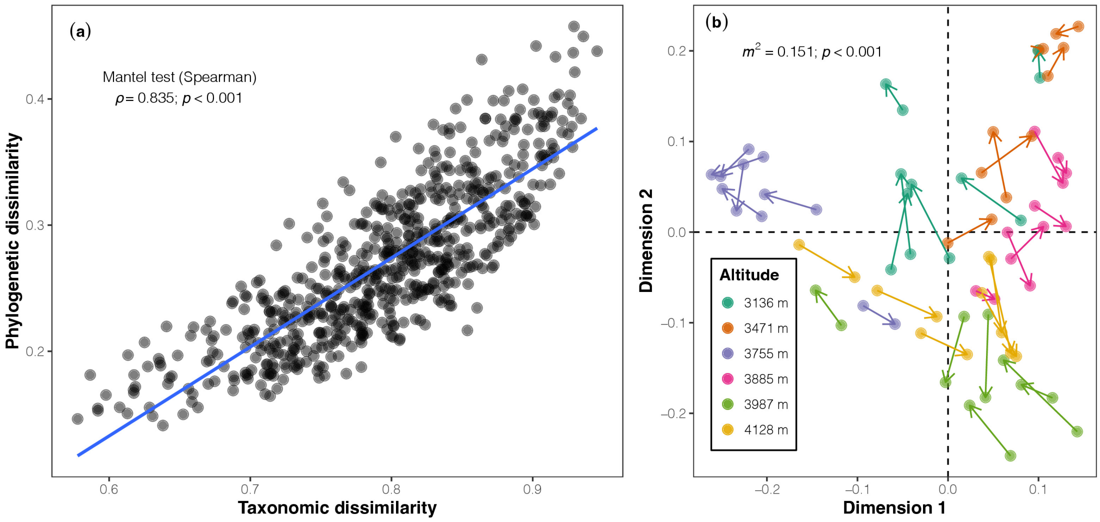

Supplementary Figure 3. Mantel correlation between soil bacterial taxonomic dissimilarity and phylogenetic dissimilarity (a) and their Procrustes plots in the ordination space (b). The inset texts show the Spearman correlation coefficient in (a) and the result of Procrustes analysis in (b), respectively.

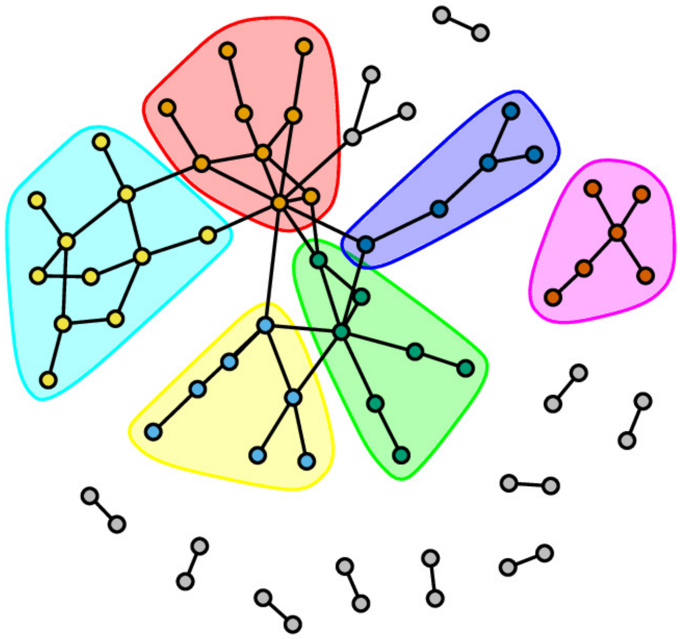

Supplementary Figure 4. The co-occurrence network of soil bacterial interactions. The network was inferred using SpiecEasi, based on ASVs present in more than half of the samples to ensure reliable association inference. Large modules containing more than six nodes are highlighted in different colors.

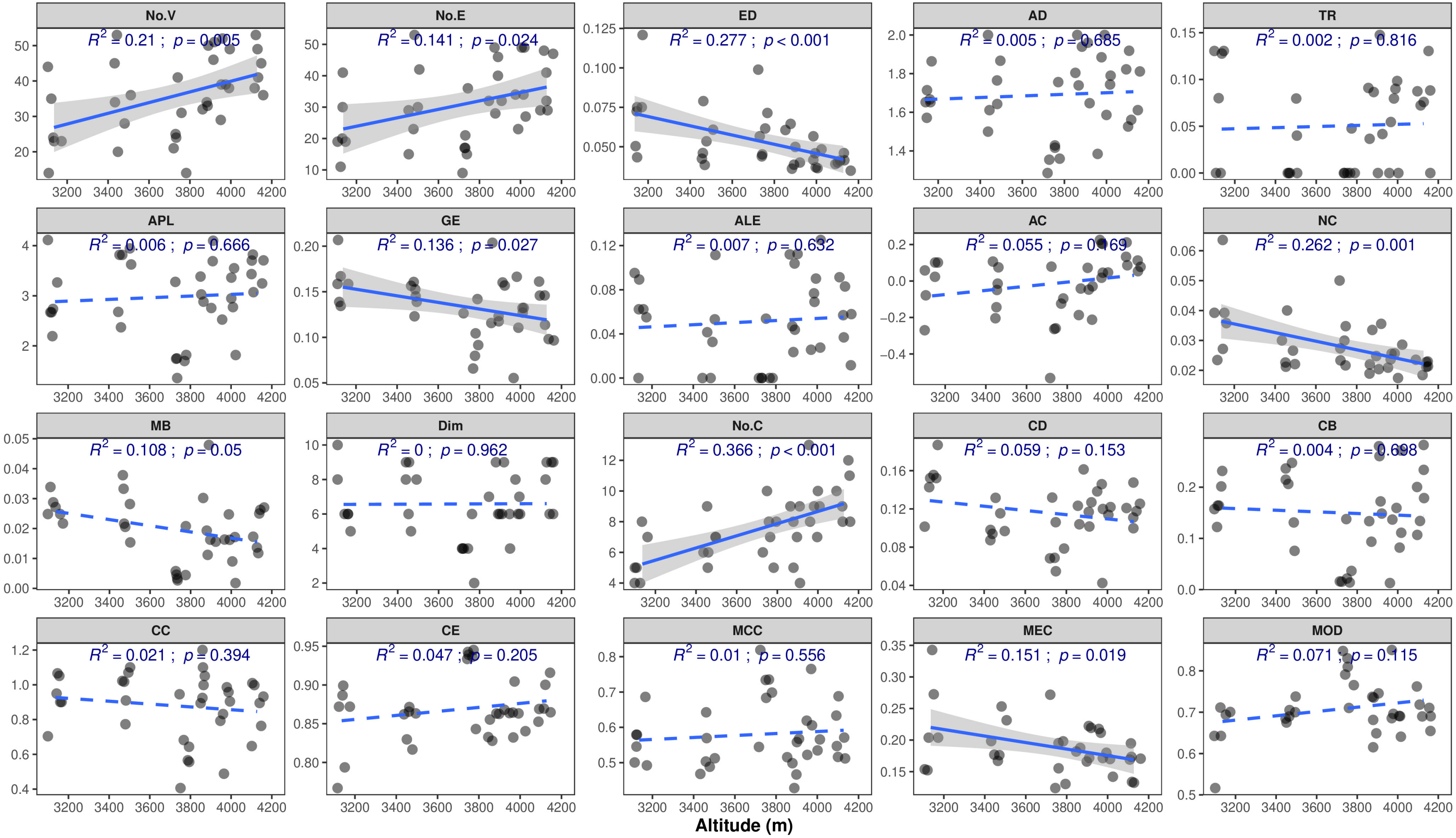

Supplementary Figure 5. Altitudinal trends in the twenty properties of soil bacterial co-occurrence network. Please refer to Supplementary Table

1 for the abbreviation of each property. Solid blue lines indicate significant relationships between each property and altitude, while the dashed lines represent nonsignificant relationships. The explained variation (R^2) and significance (p-value) are provided in each subplot.

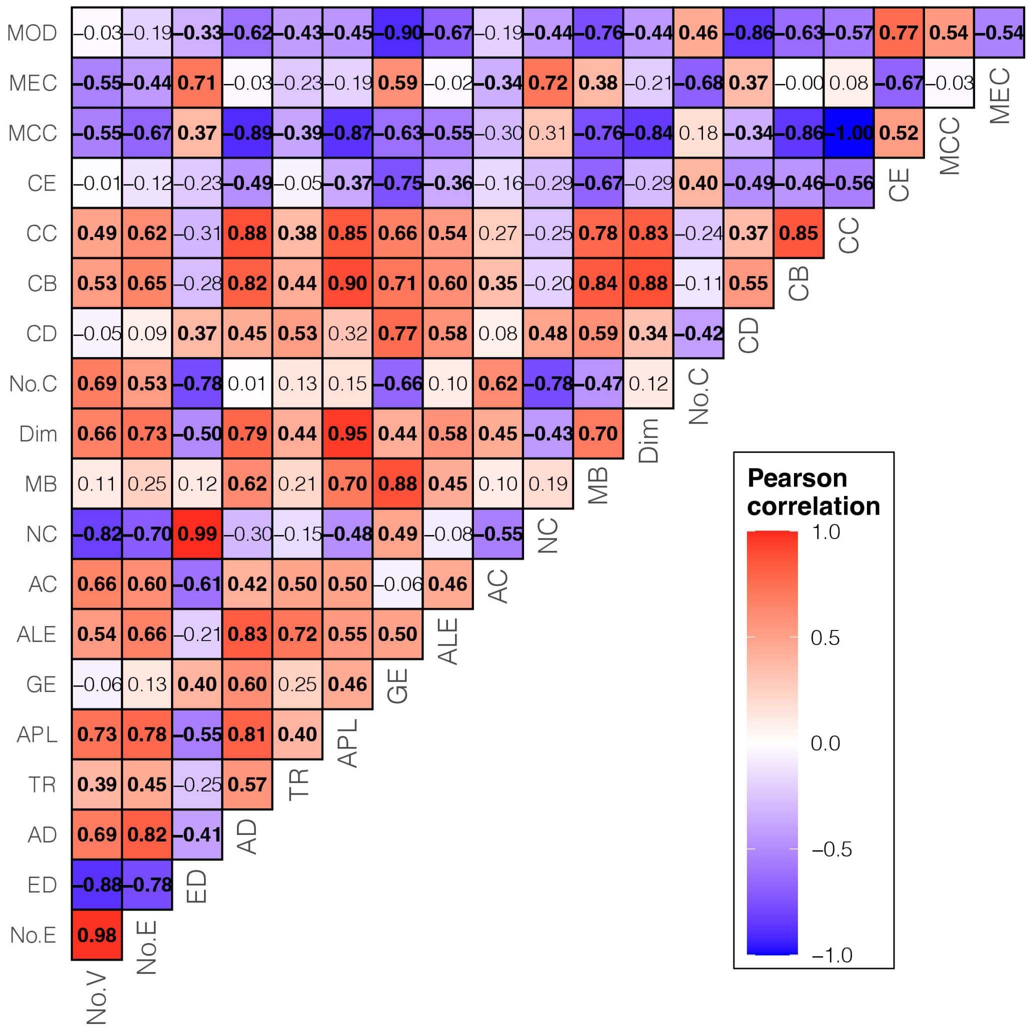

Supplementary Figure 6. Pearson correlations among the twenty properties of the soil bacterial co-occurrence networks. Please refer to Supplementary Table 1 for the abbreviation of each property. Significant correlations are shown in bold.

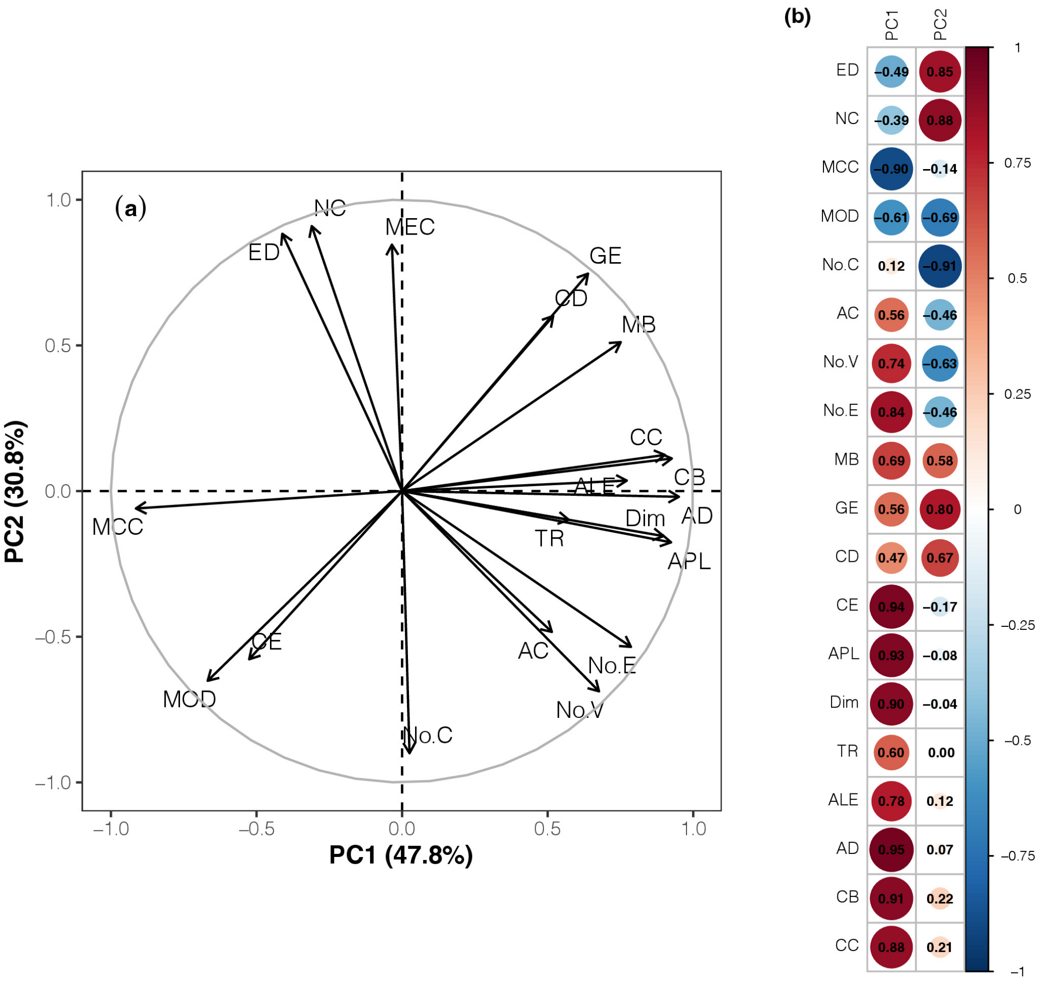

Supplementary Figure 7. Variable correlation plot of PCA results based on the twenty properties of soil bacterial co-occurrence networks (a) and the loadings of each property to the first two principal components (b). Abbreviations for each property can be found in Supplementary Table 1.

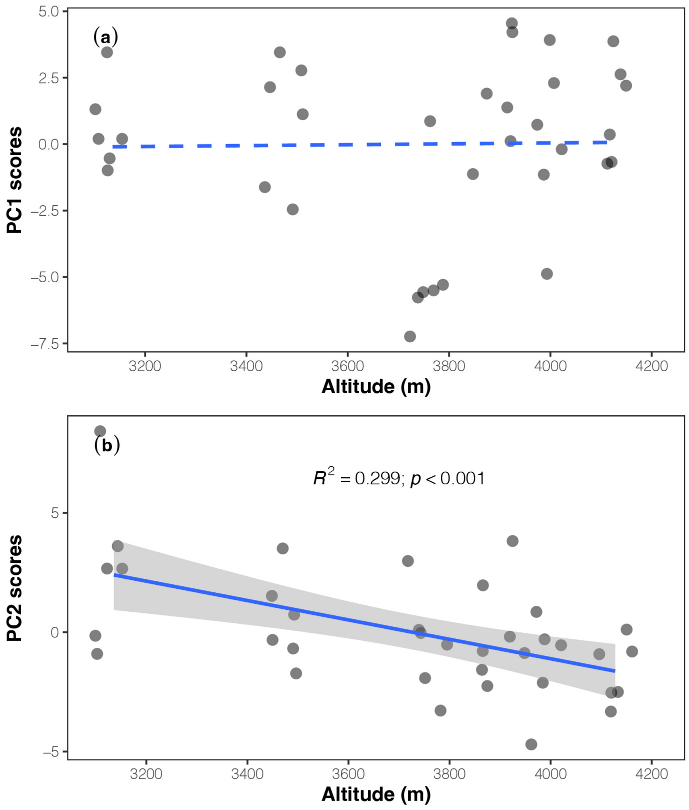

Supplementary Figure 8. Altitudinal trends in the scores of the first two principal components of PCA based on the twenty properties of the soil bacterial co-occurrence networks. The solid blue line shows a significant relationship while the dashed line represents a nonsignificant relationship.

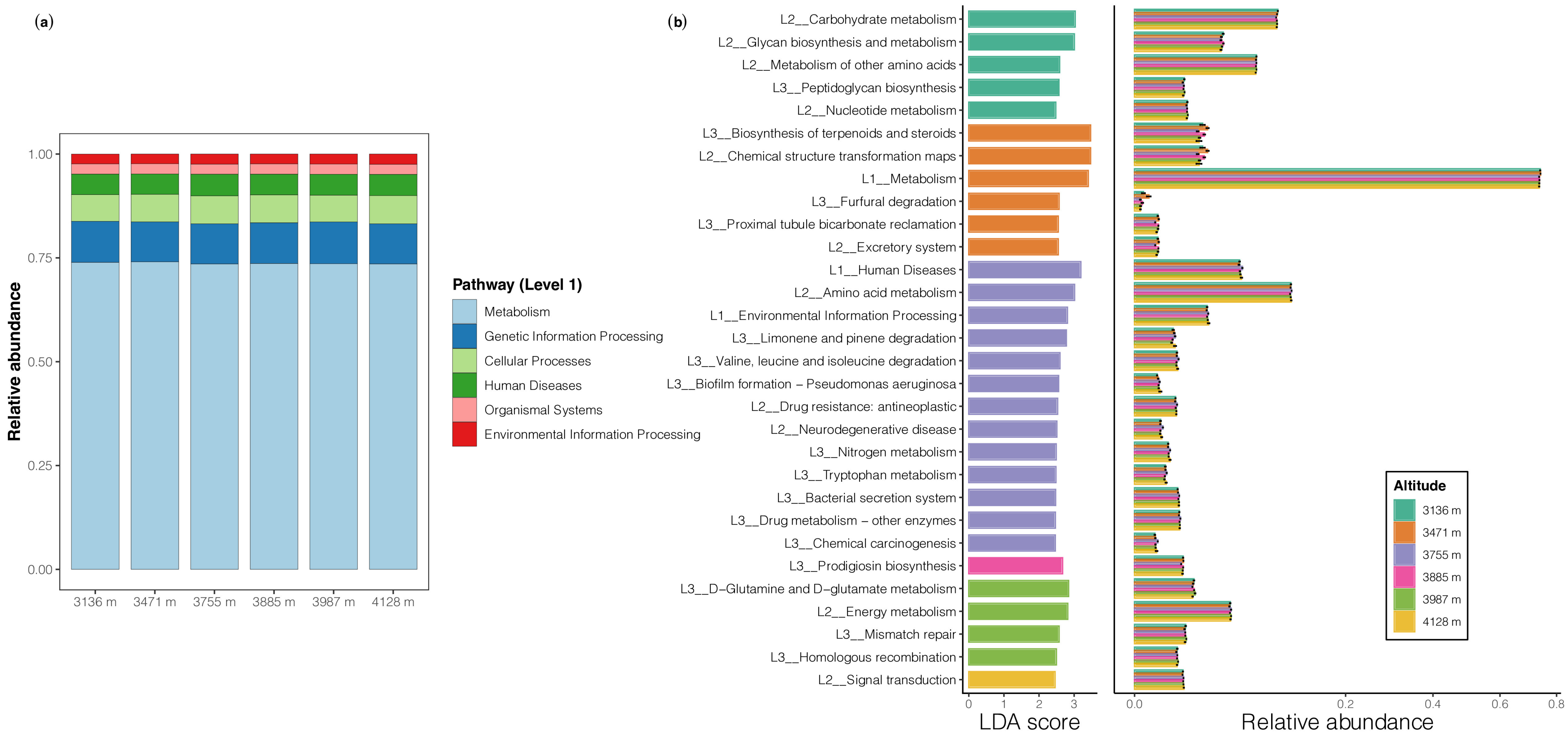

Supplementary Figure 9. The abundance and differences in KEGG pathways of soil bacterial functional potentials across altitudes. (a) Relative abundance of KEGG pathways at Level 1 based on functional predictions across altitudes. (b) KEGG pathways obtained from functional predictions that are differentially abundant across altitudes. The pathways were identified using linear discriminant analysis effect size (LEfSe). Only the top 30 pathways are shown in the figure; please refer to Supplementary Table 5 for all pathways with significantly different abundances across altitudes.

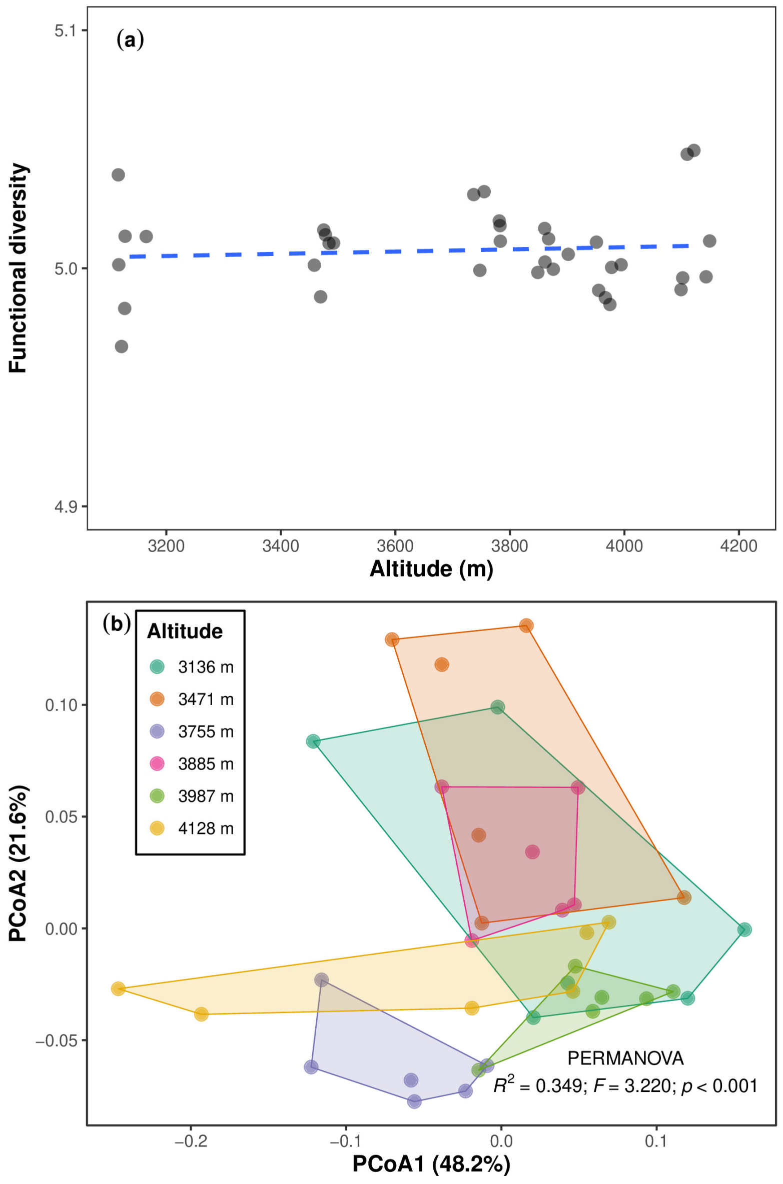

Supplementary Figure 10. Relationship between the diversity of soil bacterial functional potentials and altitude (a) and variation in composition of the functional potentials among altitudes (b). The inset text in (b) shows the results of the PERMANOVA test.

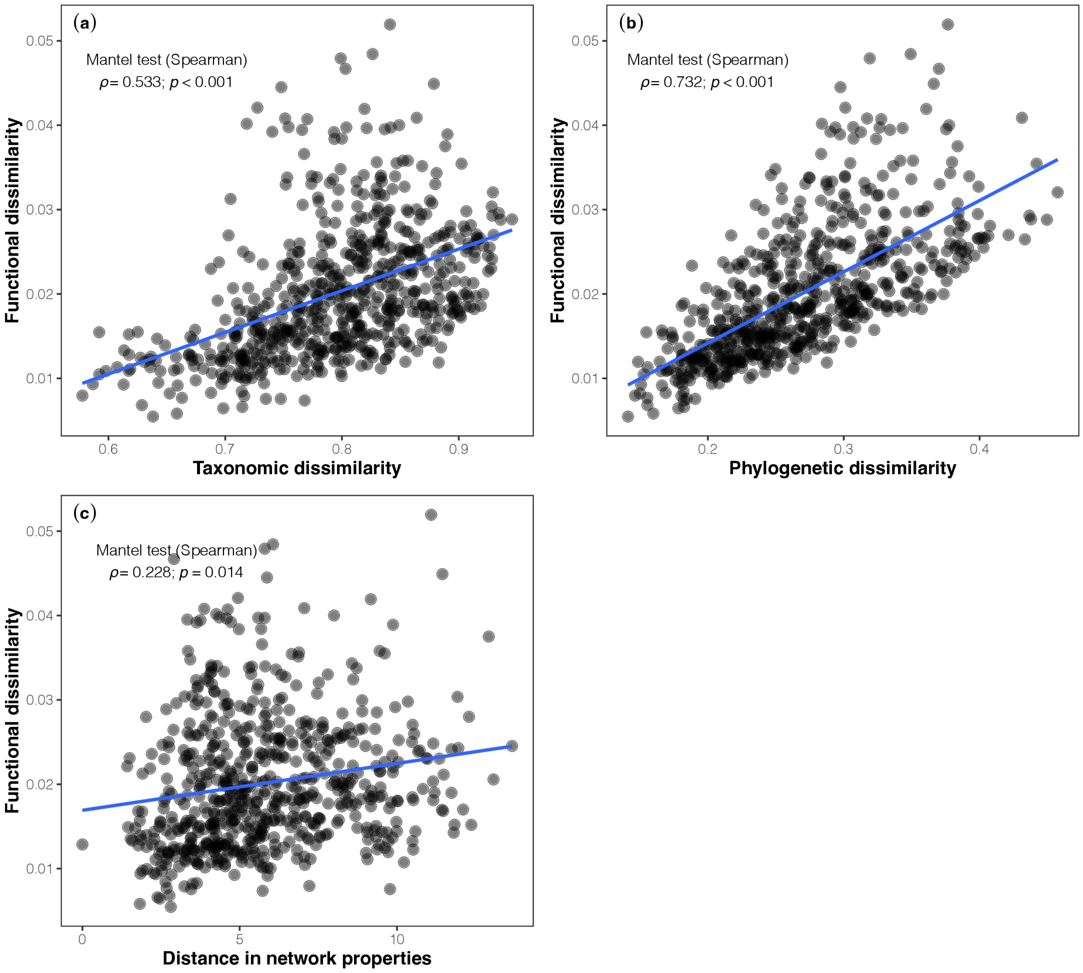

Supplementary Figure 11. Mantel correlations between dissimilarity in soil bacterial functional potentials and taxonomic dissimilarity (a), phylogenetic dissimilarity (b), and distance in co-occurrence network properties (c), respectively.
